# Technical Feasibility of Respiratory Monitoring in Dogs Using Time-of-Flight Depth Imaging

**DOI:** 10.3390/s26216986

**Published:** 2026-11-05

**Authors:** Oskar Seifert, Marcel Geis, Niklas Alexander Koehler, Manuel Stenger, Volker Groß, Martin Kramer, Keywan Sohrabi

**Affiliations:** 1 Faculty of Health Sciences, University of Applied Sciences, 35390 Giessen, Germany; marcel.geis@ges.thm.de (M.G.); n.koehler@ib-gk.com (N.A.K.); stenger@thoratech.de (M.S.); volker.gross@ges.thm.de (V.G.); keywan.sohrabi@ges.thm.de (K.S.); 2 Faculty of Veterinary Medicine, Small Animal Clinic, Justus Liebig University, 35390 Giessen, Germany; martin.kramer@vetmed.uni-giessen.de; 3 Medical Faculty, Justus Liebig University, 35390 Giessen, Germany; 4 Institute of Medical Informatics (IMI), Justus-Liebig-University, 35390 Giessen, Germany

**Keywords:** time-of-flight imaging, respiratory monitoring, dog, depth camera, signal processing, technical feasibility, method comparison

## Abstract

**Highlights:**

**What are the main findings? **

• ToF depth recordings yielded oscillatory surface distance signals in the studied canine setting.
• Agreement between camera-derived respiratory rates and concurrent manual counts varied across dogs and sessions.

**What is the implication of the main finding? **

• ROI placement and temporal processing influence rate estimates and require evaluation before clinical use.

**Abstract:**

Time-of-flight (ToF) depth imaging offers contactless observation of respiratory surface motion in veterinary patients. We investigated the technical feasibility of extracting canine respiratory waveforms and explored agreement between recorded camera-derived respiratory rates and concurrent manual counts. Depth images were acquired at a nominal 10 frames/s, and a fixed region of interest (ROI) supplied a mean depth signal. Clinical rates were obtained using Fourier spectral analysis with interpolation. Four dogs contributed eight sessions and 40 paired one-minute observations. Mean bias was +2.33 breaths/min (95% confidence interval −3.60 to +8.25), mean absolute error was 3.93 breaths/min, and exploratory repeated-measures limits of agreement were −10.40 to +15.05 breaths/min. One dog accounted for 69.4% of the absolute error and all observations above 25 breaths/min. Supplementary analyses compared spectral and peak-based estimates on identical inputs. An additional analysis of 27 ROI configurations in each of two recordings from one dog found maximum minute estimate changes of 7.50 and 7.80 breaths/min relative to a fixed baseline ROI. These findings demonstrate waveform extraction and dependence on spatial and temporal processing. They do not establish reliable respiratory rate measurement across dogs or respiratory rate ranges. The small independent sample, heterogeneous errors, incomplete original FFT configuration records and unquantified reference timing constrain the clinical comparison.

## 1. Introduction

Respiratory rate is a practical component of canine clinical observation, but its interpretation depends on the animal’s state and setting. Studies of apparently healthy dogs and dogs with subclinical cardiac disease distinguished sleeping from resting respiratory rates and used repeated one-minute counts collected at home [1,2]. Work in dogs and cats with medically controlled congestive heart failure further supports longitudinal observation under defined conditions [3]. These populations differ from hospitalized dogs. Their frequency distributions should not be adopted as universal alarm thresholds for a new monitoring system.

Manual counting requires an observer during each measurement interval. Wearable alternatives include inertial sensors evaluated for respiratory rate monitoring in dogs [4] and a wireless harness assessed in hospitalized canine patients [5]. Experimental surface contact sensors have also been used to characterize diaphragmatic timing in dogs [6]. These approaches measure related but distinct physiological or mechanical signals, and their agreement with a chosen reference must be established for the intended application.

Contactless animal studies have investigated ultra-wideband radar in dogs and cats [7] and infrared thermography in anesthetized pigs [8]. Together, these studies motivate remote observation while illustrating how species, posture, movement, reference modality and acquisition conditions define the scope of any validation.

Time-of-flight (ToF) cameras estimate a distance at each image location. Respiratory expansion and contraction change the distance to the visible thoracoabdominal surface, producing a depth signal over time. Early ToF work derived regional respiratory signals for image gating [9] and modeled torso surfaces to estimate respiratory motion [10]. Later Kinect and RGB-D studies investigated respiratory rate from chest distance changes [11] and visual/depth signal analysis [12]. Mean depth aggregation with automatically located body regions has also been demonstrated [13]. Thus, regional depth aggregation has an established methodological basis. Its performance in hospitalized dogs remains a separate question.

The choice of region of interest (ROI) and processing method affects the relationship between the observed surface and breathing. Thoracic regions do not necessarily move identically [14], and multi-camera chest wall analysis [15] or comparison with respiratory inductance plethysmography [16] assesses information beyond a single mean depth waveform. A parameter study compared mean, median, principal component and motion reference approaches across different human acquisition conditions [17]. Camera characterization additionally demonstrates sensitivity to geometry, reflectivity and infrared illumination [18], as well as ToF-specific noise behavior [19]. These findings motivate explicit reporting of both acquisition and signal processing.

The evidence can be compared using consistent criteria. In 19 hospitalized dogs, Warhoe et al. compared a wearable harness with manual 30 s thorax counts scaled to a minute. Respiratory rate limits of agreement exceeded their prespecified ±6 breaths/min criterion, and continuous output availability was not reported [5].

Addison et al. studied seven healthy adults across 553 segments using capnography or spirometry, prescribed rates of 4–40 breaths/min, and varied posture, lighting and coverings. They reported a root mean square deviation of 0.69 breaths/min and 99.9% uptime under those conditions [20].

In a controlled comparison of six depth signal extraction variants, Kempfle and Van Laerhoven examined 19 adults and 380 recordings with varied poses, occlusions and distances. The thoracic Diff Model had a mean frequency error of 0.3 breaths/min against a pacing target, rather than an independent respiratory recording, and clinical continuous availability was not reported [17]. Valenzuela et al. studied ten participants with paced breathing and a separate group of 20 with a respiratory belt during static indoor measurements. Their thoracic comparison reported a bias of 0.07 breaths/min and explicitly 96% limits of agreement of −2.21 to +2.34 breaths/min, without a comparable continuous availability measure [13]. These results support depth sensing under their tested conditions but do not resolve performance in moving, fur-covered canine patients.

Recent clinical studies extend this evidence beyond controlled resting measurements. MacLeod et al. evaluated 34 adult volunteers during anesthesia using a combined capnography and impedance reference. Their overall RMSD was 1.92 breaths/min and bias 0.30 breaths/min, with analysis restricted to periods of agreement between the two reference devices [21]. This illustrates both the value of instrumental references and the influence of eligibility rules on reported performance.

Chavernac et al. combined automated infrared ROI detection with pixel-wise volume estimation and evaluated their system in a healthy adult and a critically ill child [22]. Kojima et al. compared anatomically defined torso regions against a respiratory belt waveform in seated participants and found the most stable waveform estimates in the chest region under their tested conditions [23]. These studies motivate anatomical ROI evaluation and waveform-level reference measurements. Their human localization models and acquisition conditions require separate assessment before application to canine anatomy, fur and posture.

Evidence remains limited for the performance of regional ToF respiratory sensing in hospitalized dogs, particularly for the influence of processing choices and variability across sessions. We investigated whether our ToF system could extract interpretable respiratory motion signals during routine observation of hospitalized dogs and how its respiratory rate estimates agreed with concurrent manual counts. The scientific contribution is the characterization of canine depth signals, the analysis of processing dependence on identical recordings, and the quantification of agreement at dog and session level. We applied established signal processing methods to this veterinary setting. The clinical comparison and supplementary signal analyses address complementary questions about practical feasibility and estimator behavior.

Accordingly, the study had two aims: (1) to characterize respiratory motion waveforms extracted from regional ToF depth recordings in hospitalized dogs, including their sensitivity to processing choices, and (2) to explore agreement between the recorded camera-derived respiratory rate estimates and concurrent manual counts. The contribution is an application-specific characterization of the method and its limitations. Section 2 describes the study design, acquisition and analyses. Section 3 reports the waveform and agreement results. Section 4 and Section 5 interpret the findings and requirements for further validation.

## 2. Materials and Methods

### 2.1. Study Design and Reference Measurements

**Figure 1:**
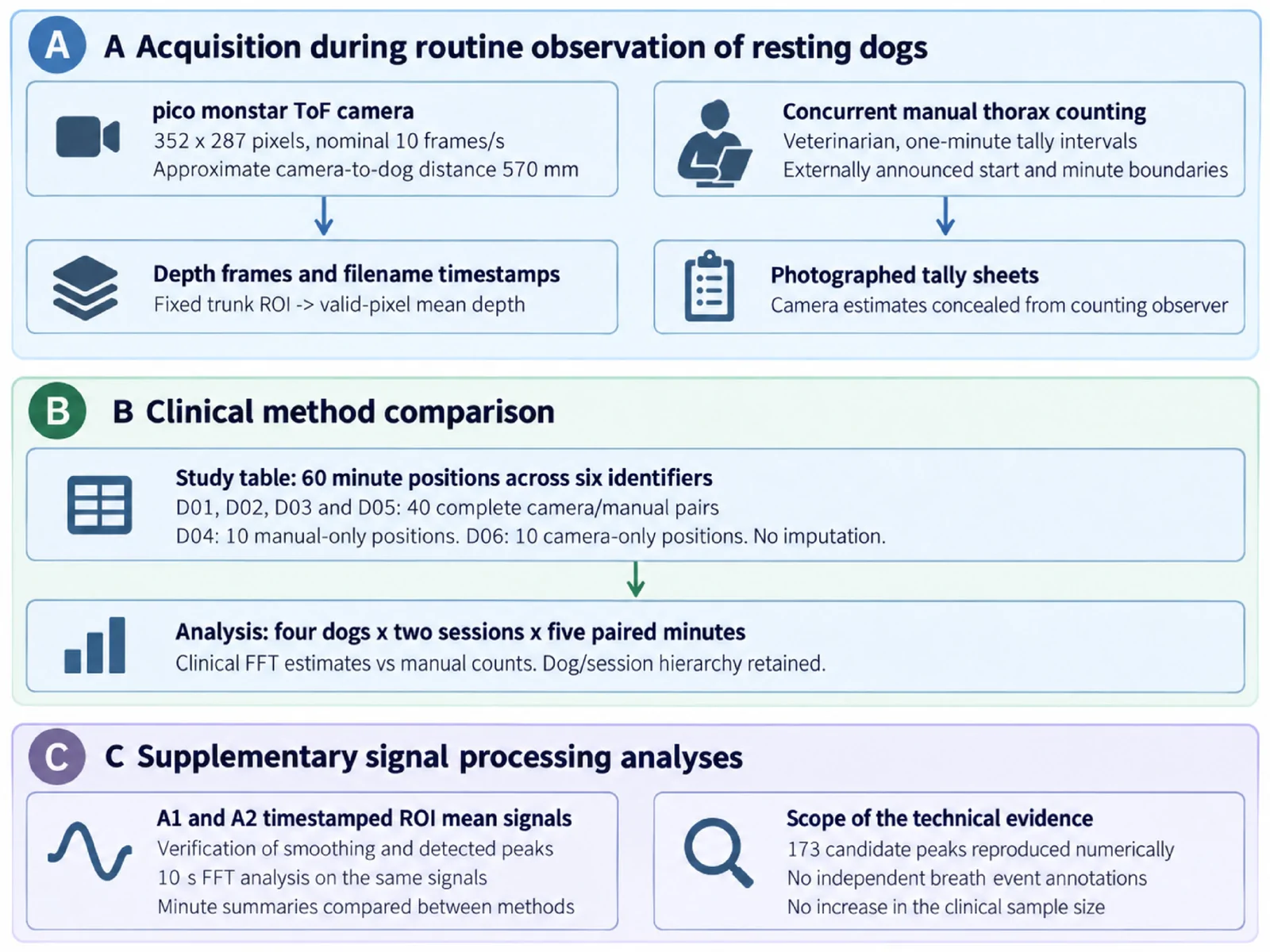
Study design, acquisition and analysis. Camera-derived FFT estimates and concurrent manual counts form the clinical comparison. The recordings A1 and A2 provide the inputs for supplementary signal processing analyses. Acquisition and counting were coordinated prospectively. Residual interval error was not quantified. The 60 table positions describe the results dataset rather than all attempted acquisition time.

**Figure 2:**
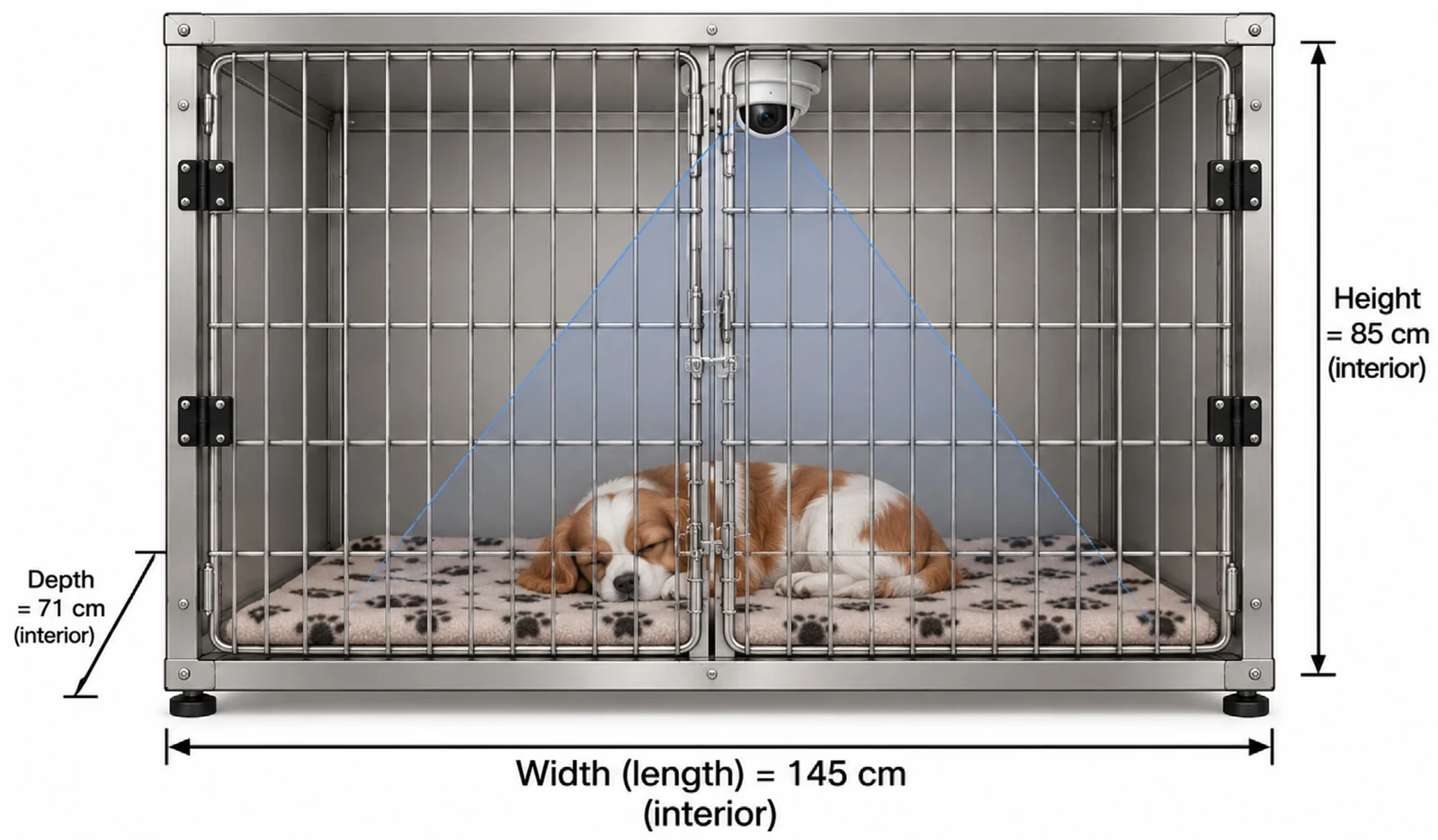
Schematic of the recording enclosure and camera placement. The illustrated enclosure has interior dimensions of 145 × 71 × 85 cm. The camera was mounted centrally at the top using a flexible mount. The illustrated viewing lines are schematic and do not represent a calibrated field-of-view measurement.

We conducted this technical feasibility study during routine observation of hospitalized dogs in Giessen, Germany, between July 2021 and March 2022. Measurements targeted resting dogs. Substantial movement, restlessness or panting that interfered with respiratory signal extraction precluded use in the paired analysis. Selection was qualitative, without a numerical motion threshold or a complete log of attempted and rejected recordings. We evaluated agreement with manual counting and performed supplementary analyses of the respiratory waveforms and processing methods. Figure 1 summarizes the study design, while Figure 2 shows a schematic of the recording enclosure and camera placement.

The analyzed sample comprised four dogs: a female short-haired Dachshund mix (12.0 kg, 6 months old), a female long-haired Cairn Terrier (6.8 kg, 1 year 3 months old), a female long-haired German Shepherd mix (13.4 kg, 11 years 6 months old), and a male short-haired Terrier mix (18.4 kg, 12 years 11 months old). Body condition scores, quantitative thoracic conformation measurements and standardized clinical condition categories were not collected.

No formal a priori sample size calculation was performed. This was a convenience sample obtained during routine clinical observation and the study was designed as a technical feasibility assessment. The four dogs are the independent biological units. The 40 min level pairs are repeated observations and do not increase the independent sample size. A follow-up study to expand the dataset is planned.

Recordings were not retained when movement, turning/restlessness or panting interfered with observation. These decisions were qualitative, without a numerical movement threshold or formal exclusion checklist. The records did not consistently document who made each exclusion decision. The camera-derived estimate was concealed from the veterinarian performing the manual count. The exact number of rejected attempts was not logged. We therefore cannot provide a complete attempted-versus-analyzed flow diagram or quantify the effect of exclusions. Practical feasibility in this study concerns signal extraction from retained recordings. The 40 complete pairs among 60 table positions describe table completeness only, not acquisition success or signal availability.

Concurrent direct manual breath counting served as the operational reference. A veterinarian observed the thorax of each dog and counted each breath using a manual tally sheet during the defined one-minute observation intervals.

Manually counted breaths were documented on handwritten tally sheets and photographed. All 40 values included in the paired analysis agreed with these contemporaneous annotations. The wider results table contained 60 min positions across six study identifiers. D01, D02, D03 and D05 each provided two five-minute sessions with both modalities. D04 had ten manual values without camera estimates, and D06 had ten camera estimates without manual values. The 20 incomplete positions were excluded without imputation. Thus, the complete-case dataset contains four independent dogs, eight sessions and 40 repeated pairs. The table does not enumerate all acquisition attempts and cannot supply a monitoring availability denominator.

### 2.2. Camera Acquisition and Temporal Alignment

Depth images were acquired with a pico monstar 105 ToF camera (pmdtechnologies, Siegen, Germany). We developed the acquisition software using the Royale SDK (Ver.: 3.34.0.132). The study acquisition settings were FilterLevel 200 (Legacy) and UseCase MODE_9_10FPS_720, with a nominal frame rate of 10 frames/s and a resolution of 352 × 287 pixels. The camera was positioned approximately 570 mm from the resting dog. Depth images were exported as TIFF files for analysis. Application and SDK version identifiers were not recorded separately for every session.

The camera was mounted centrally at the top of the rectangular enclosure, using a flexible mount attached to the enclosure bars. The USB cable ran to a laptop outside the enclosure. Dogs generally lay on their side on a thin blanket. The enclosure schematic gives interior dimensions of 145 cm (width), 71 cm (depth) and 85 cm (height), and illustrates the central camera position. The camera-to-dog distance was approximately 570 mm as reported for the setup. Exact camera height relative to the dog, pitch angle, the reference points used for the approximate distance, ambient illuminance, infrared interference and the dog’s pixel dimensions in the image were not recorded and cannot be reconstructed reliably.

The indirect ToF principle estimates distance from the phase relationship between emitted modulated infrared illumination and reflected light [19]. Distance estimation preceded the MATLAB (Ver. R2018a) analysis, which operated on depth estimates exported as TIFF images. We used the depth channel for respiratory signal extraction. The companion infrared channel was not included in this analysis.

Depth files were selected by the prefix Depth_ and the TIFF extension. The numeric suffix of each filename was interpreted as elapsed milliseconds. The processing script converted these values to seconds and formed t(k) = T(k)/1000 − T(1)/1000 + 1/f_s_, where T(k) is the filename timestamp and f_s_ = 10 frames/s. The added sampling interval places the first processed sample at 0.1 s. Peak timing in the main script uses this timestamp-derived vector, whereas its smoothing span is specified in samples.

A timekeeper coordinated acquisition and manual counting by announcing the start, each minute boundary and the end of the five-minute observation. A second operator started the camera software, and a veterinarian counted visible thoracic movements. Camera-derived values were concealed from the counting observer. The acquisition procedure included an approximately 5 s software start lead before counting and a synchronization signal. We did not record a complete mapping of these events to the camera timestamps, so the residual alignment of individual minute intervals could not be quantified. The software start lead and an approximately 1 s monitor-to-laptop clock difference observed in a separate session describe different timing relationships. We therefore applied no common retrospective time shift.

### 2.3. Extraction of the Respiratory Depth Signal

**Figure 3:**
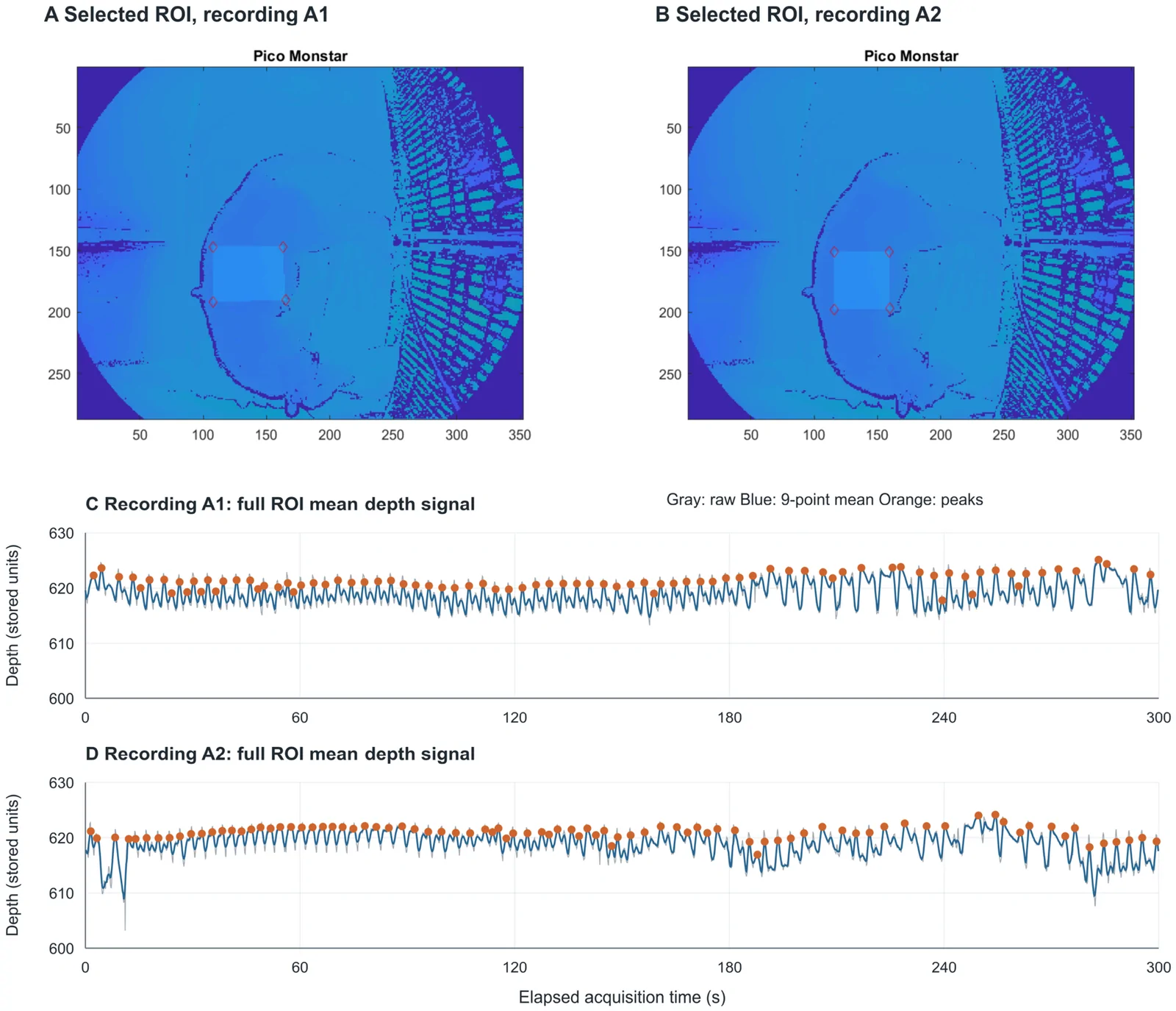
Depth images and respiratory signals from study recordings A1 and A2. (**A**,**B**): Depth image displays with four ROI vertices and the highlighted polygon. Image axes are pixel coordinates, and display colors match the original study images. (**C**,**D**): Complete raw ROI means (gray), stored nine-sample moving averages (blue) and stored detected peaks (orange). Values are stored depth units.

We selected the ROI interactively on the first depth image using Single_ROI_Mean_Analyse.m. The script supported either the full image or a polygon defined by four manually selected vertices. For regional analysis, the operator selected a visible trunk region and retained its polygon throughout the sequence. Figure 3 shows the selected regions in A1 and A2. The protocol did not specify a reproducible thoracic-versus-abdominal landmark rule, and ROI area, vertices and selector identity were not recorded systematically for each session. Our MATLAB implementation used a fixed mask without automatic tracking or quantitative motion rejection. Repeated selections by different operators were not collected. We subsequently assessed position and size sensitivity using a fixed set of new rectangular ROIs in A1 and A2, as described in Section 2.7. This additional analysis did not reconstruct the original polygons or measure interoperator repeatability.

Each image was converted to double precision. For frame k, only pixels within the fixed ROI and within the inclusive numerical depth range 1 to 2000 were retained. Other pixels were assigned NaN and excluded from the spatial mean. If D(u,v,k) denotes the depth at image coordinates (u,v), Ω the selected ROI and V(k) the retained pixels, the depth signal was

(1) *V_k_* = {(*u*,*v*) ∈ *Ω* : 1 ≤ *D*(*u*,*v*,*k*) ≤ 2000}

(2) $$s_{k}=\frac{1}{\left|V_{k}\right|}\sum_{(u,v)\;\in \;V_{k}}D(u,v,k)$$

The exported depth scale was one stored unit per millimeter. No additional unit conversion was applied in MATLAB. The retained pixel set could vary between frames when values crossed the validity limits. The implementation did not impose a minimum valid-pixel fraction or a separate rule for a completely invalid ROI.

This operation produces a scalar time series of mean ROI depth. It does not establish correspondence between physical surface points in successive frames, reconstruct metric three-dimensional trajectories, or estimate displacement vectors for individual pixels. Respiratory motion is represented by changes in the aggregate camera-to-surface distance. Spatial averaging can reduce uncorrelated pixel noise, while gross motion, occlusion or changes in the retained surface can also alter the resulting signal. This differs from RGB feature tracking with corresponding depth measurements, as implemented in a multimodal breathing monitor [24].

### 2.4. Spectral Estimation of Respiratory Rate

We estimated clinical respiratory rate from the dominant periodic component of the ROI mean depth signal using an FFT with spectral interpolation. Our spectral implementations used mean subtraction, Hann windowing and zero padding. During development, we implemented variants with different segment definitions and time resampling. We describe these variants in Supplementary Section S6. Session-specific processing configurations and the aggregation of short-window estimates were not logged with every clinical minute value, which limits exact regeneration of the 40 clinical estimates. The agreement analysis therefore uses the clinical values recorded in the study results table.

For an N-sample, uniformly sampled analysis segment x(n), the discrete Fourier transform underlying this estimator can be written as follows:

(3) $$(m)\;=\;\sum_{n\;=\;0}^{N\;-\;1}x(n)\;\exp(-i\;2\mathrm{\pi mn}/N),\;f_{m}\;=\;m\;f_{s}/N$$

Here, f_s_ is the sampling frequency, i is the imaginary unit and m indexes frequency bins. A Hann window reduces spectral leakage at the cost of a wider spectral main lobe [25]. Zero padding samples the finite-record spectrum on a denser frequency grid without adding independent information or extending the observation duration. The selected frequency f^ is converted to breaths per minute as follows:

(4) $${\mathrm{RR}}_{\mathrm{spectral}}\;=\;60\;\hat{f}$$

Our first spectral implementation analyzed nonoverlapping segments of 50 samples, subtracted their mean, applied a Hann window and zero-padded to NFFT = int(f_s_/0.005). A second implementation used nonoverlapping 10 s segments and linearly resampled the timestamped signal before applying the Hann window. Both selected a local magnitude maximum in the positive spectrum and rounded frequency × 60 to an integer. These FFT routines did not apply the nine-sample moving average of the separate MATLAB peak analysis or an explicit respiratory band restriction. In our code, interpolated_fft refers to zero padding, whereas linear interpolation refers to resampling the signal in time. Neither operation denotes a parabolic spectral peak fit.

For the supplementary comparison of spectral and peak-based estimates, we analyzed A1 and A2 using the same raw ROI means and acquisition-relative intervals. We linearly interpolated the signals at 10 Hz from 0.0 to 299.9 s without extrapolation. Each recording supplied 30 nonoverlapping 10 s windows of 100 samples. We subtracted each window mean, applied a symmetric Hann window and calculated a 2000-point real FFT with frequencies k × 10/2000 Hz. The largest magnitude within 0.10–1.50 Hz was selected, including the boundaries. This analysis used no moving average smoothing, band-pass filter, quality rejection or additional peak refinement.

The unpadded bin spacing was 0.1 Hz (6 breaths/min), and the zero-padded frequency grid was 0.005 Hz (0.3 breaths/min). We summarized each minute by the median of six unrounded 10 s estimates. In the matching intervals [0,60), [60,120), [120,180), [180,240) and [240,300) s, the peak estimator used 60 divided by the median interval between consecutive peaks within that minute. Parameters were not tuned to the manual counts. This supplementary analysis compares estimator behavior on identical inputs. It does not replace the original clinical estimates, because exact correspondence to the manual interval boundaries was not documented for A1 and A2. Code S1 and Data S1 contain the executable implementation, parameters and window-level results.

### 2.5. Smoothing and Peak Detection in the MATLAB Time Domain Workflow

The main workflow applies smooth(s,10) to the ROI mean signal. With finite input data, the default operation is a moving average. MATLAB reduces an even moving average span by one, so the requested span of 10 corresponds to an effective nine-sample window. At the nominal frame rate, this is approximately 0.9 s by sample count convention, with 0.8 s between the first and last sample centers in an interior window. The implementation is centered offline smoothing. Real-time processing latency was not evaluated.

(5) $$\tilde{s}(k)=\frac{1}{9}\sum_{j=-4}^{4}s(k+j)$$

For interior samples, this centered mean combines four preceding values, the current value and four following values. At the beginning and end of the recording, symmetric windows shorten to 1, 3, 5 and 7 samples. This boundary rule was verified against the saved numerical output. Mean depth oscillations are plotted with their original sign: expansion toward the camera can reduce distance, so detected positive maxima are cycle landmarks rather than confirmed inspiration onsets.

Local maxima were identified in the smoothed signal using findpeaks with the timestamp vector and a minimum peak prominence of 1.5 depth units. Prominence describes the elevation of a peak above its surrounding baseline and differs from an absolute peak height threshold. The main script imposes no minimum interpeak distance. Detected peaks therefore represent candidate respiratory cycles, without a separate automated classification of gross body movement or other non-respiratory disturbances.

The recording-level respiratory rate estimate was computed from the median interval between successive detected maxima:

(6) $${\mathrm{RR}}_{\mathrm{interval}}\;=\;\frac{60}{\mathrm{median}[\tau_{j+1}\;-\;\tau_{j}]}$$

Here, τ(j) denotes a detected peak time in seconds. This estimator requires at least two detected peaks and summarizes typical cycle spacing over the analyzed recording. It is not identical to counting all breaths within a fixed minute, particularly when breathing is irregular, pauses occur or boundary cycles are included differently. The code prints the estimate with two significant digits. That display precision is distinct from measurement accuracy. Our development FFT routines round frequency × 60 to the nearest integer. Clinical estimates were analyzed at their recorded integer precision, because unrounded outputs and the export configuration were not saved. Nearest integer rounding changes an estimate by at most 0.5 breaths/min. The two-significant-digit MATLAB display is a separate formatting operation.

### 2.6. Experimental Baseline Filtering and Time Binning

Median_Baseline_Filter.m contains a separate exploratory processing branch. A 50-sample moving median is subtracted from the unfiltered ROI mean to remove slow baseline variation. The residual is then smoothed using the same nominal span of 10 samples. Final peak detection uses a minimum prominence of 2 depth units and a minimum separation of 2.5 s, beginning at sample 7. The median filter call uses the function’s default boundary treatment, which includes zero padding. Omitting early samples from the final peak search is an implemented choice. It does not by itself establish that all edge effects have been eliminated.

(7) *b_k_* = *median*_50_(*s*)(*k*), *r_k_* = *s_k_* − *b_k_*

The 2.5 s minimum separation limits the density of accepted peaks to approximately 24 per minute in sustained periodic breathing. It is therefore unsuitable, without adjustment, for resolving every cycle at the higher manually observed rates in this dataset. This parameter belongs to the experimental branch and should not be interpreted as the verified setting used for all summary table values.

Calculate_BPM.m uses the experimental peak times to construct a histogram. Recording length is estimated as frame count divided by the nominal frame rate, and peak times are multiplied by that rate before binning. Full bins correspond nominally to 60 s. The final partial bin contains an unnormalized count. A count C over an interval of duration Δt seconds would require the rate conversion 60C/Δt, which this script does not implement for the partial bin. These settings characterize the experimental code and are not a validated parameter choice for the full clinical rate range. Table 1 summarizes the MATLAB branches.

### 2.7. Waveform Verification and Processing Diagnostics

We performed supplementary waveform analyses on two study recordings acquired on 28 March 2022, designated A1 and A2. Both originated from the D05 measurement session. We used the timestamps, raw ROI means, smoothed means and peak coordinates saved in our native MATLAB figures, together with the corresponding depth image sequences. This preserved the exact numerical inputs used in our time domain processing. ROI vertices had not been saved separately, so the original waveform verification used the stored ROI signals. The new ROI sensitivity analysis below used explicitly defined rectangles and freshly extracted signals. The recordings contribute technical observations within the existing study sample.

We verified the main time domain workflow by recalculating the nine-sample centered mean with shortened endpoint windows and local maxima at prominence ≥1.5. We then applied our experimental baseline-filtering branch to the same raw ROI means. A zero-padded 50-sample median, comprising samples k − 25 through k + 24 and averaging the central order statistics, was subtracted from each signal. The residual underwent nine-sample smoothing, prominence-based peak detection at ≥2 and a minimum separation of 2.5 s. Peak selection began at sample 7 and used timestamp separation, retaining the higher peak when candidates conflicted. Applying both branches to identical inputs avoids differences caused by the ROI traces used in the original development runs.

For visual comparison, the same elapsed-time interval, 60–120 s, was displayed for both recordings and both branches. Parameter sensitivity was assessed over all 20 combinations of smoothing spans 3, 5, 9 and 15 samples and prominences 0.5, 1, 1.5, 2 and 3 depth units, without a minimum peak separation. The total number of candidate peaks was recorded for each complete recording. This analysis characterizes processing sensitivity and did not select settings by minimizing error against manual counts. Separating respiratory signal extraction from rate estimation is also useful in comparative algorithm evaluation using other physiological signals [26].

The theoretical amplitude response of an M sample rectangular moving average was calculated for a nominal sampling frequency f_s_ = 10 Hz:

(8) $$\left|H(f)|=|\frac{\sin(\mathrm{\pi Mf}/f_{s})}{M\;\sin(\mathrm{\pi f}/f_{s})}\right|$$

This response describes interior uniformly sampled windows, not endpoint behavior or the nonlinear median filter. To examine the observed frequency content without introducing a resampling step, normalized Lomb–Scargle periodograms were calculated from mean-centered raw ROI signals at the original timestamps. Frequencies covered 0.10–1.50 Hz in 0.001-Hz steps. Spectral peaks were treated as descriptive features, without a significance test or an assumption that the strongest spectral peak must equal the respiratory fundamental. We used the Lomb–Scargle formulation for unevenly spaced observations [27].

We performed an additional retrospective ROI sensitivity analysis using all 2964 A1 and 2959 A2 depth frames. Both recordings came from D05. Before calculating the ROI-dependent outcomes, we specified a 40 × 40-pixel baseline rectangle with zero-based columns 120–159 and rows 150–189 on the first depth images. We tested the full factorial combination of side lengths 32, 40 and 48 pixels and horizontal and vertical shifts of −4, 0 and +4 pixels. The 27 fixed rectangles per recording varied the side length by 20% and position by 10% of the baseline side length. These new regions were not claimed to reproduce the original manually selected polygons. Coordinates and all outcomes are supplied in Supplementary Materials S2 and Data S2.

For each ROI, we recomputed the mean of depth pixels within 1–2000 inclusive at every original frame timestamp and applied the unchanged supplementary FFT estimator from Section 2.4. All configurations and all 30 short windows per recording were retained. We described valid-pixel fractions, waveform correlation with the baseline ROI, mean-subtracted waveform differences, and changes in the 10 s and minute-level spectral estimates. Correlation characterizes waveform similarity rather than physiological accuracy. No ROI or spectral parameter was selected by minimizing disagreement with the manual counts. The 26 nonbaseline comparisons per recording are repeated perturbations of one signal, not independent biological observations. We therefore report descriptive ranges without population confidence intervals or hypothesis tests. Code S2 reproduces extraction from the depth images and all ROI-dependent estimates.

### 2.8. Statistical Analysis of Agreement

We analyzed the 40 clinical minute-level camera estimates against their concurrent manual counts. The clinical rates were obtained by dominant frequency estimation with spectral interpolation. The supplementary waveform and FFT analyses used A1 and A2 to evaluate processing behavior separately from this paired clinical comparison.

For each paired observation i, the signed difference was d(i) = RRToF(i) − RRmanual(i). The mean signed difference, mean absolute error (MAE) and root mean square error (RMSE) were calculated as follows:

(9) $$\mathrm{Bias}=\frac{1}{n}\sum_{i=1}^{n}d_{i},\mathrm{MAE}=\frac{1}{n}\sum_{i=1}^{n}\left|d_{i}\right|,\mathrm{RMSE}=\sqrt{\frac{1}{n}\sum_{i=1}^{n}{d_{i}}^{2}}$$

The range of differences and the proportions within 2 and 5 breaths/min are reported as descriptive summaries. These tolerances were not prespecified clinical acceptance criteria. Agreement was evaluated from paired differences rather than correlation [28,29]. The dog was the independent biological unit. All available paired values were retained, including the largest deviations. Missing reference values were not imputed.

To account for the balanced structure of four dogs, two sessions per dog and five minutes per session, a nested random intercept model was fitted to the differences:

(10) *d_ijk_* = *μ* + *u_i_* + *v_ij_* + *ε_ijk_*

Here, μ is the mean bias, u(i) the dog effect, v(i,j) the session-within-dog effect and ε(i,j,k) the residual. The model assumes mutually independent, zero-mean normal effects with variances σ^2^dog, σ^2^session and σ^2^residual. Variance components were estimated by balanced nested analysis of variance. All three were positive in the observed data, making these estimates equivalent to interior restricted maximum likelihood estimates. This implements the repeated-measures agreement principle for a varying true respiratory rate, extended to include session variability [30,31].

(11) $$\mathrm{LoA}=\mu \pm 1.96\sqrt{{\sigma_{\mathrm{dog}}}^{2}+{\sigma_{\mathrm{session}}}^{2}+{\sigma_{\mathrm{residual}}}^{2}}$$

The resulting limits concern a single paired minute from a new dog/session under the modeled conditions, rather than a mean over ten minutes. The 95% confidence interval for bias used the four dog-specific mean differences, their standard error and the t distribution with three degrees of freedom [32]. Because all dogs contributed ten pairs, the equally weighted dog mean equals the pooled mean.

Uncertainty in each limit of agreement was quantified using 50,000 parametric bootstrap-t replicates, simulating all three hierarchical levels and refitting nonnegative variance components for each replicate (seed 20260911). Studentization used the variance of the fitted bias and a delta method standard error for the total standard deviation. The balanced mean square derivation is supplied in the statistical supplement. This is a model-based extension of published bootstrap agreement methods [31,32]. Confidence intervals for the limits quantify estimation uncertainty and are distinct from the limits themselves [33].

Differences by dog, session and minute, conditional residuals against fitted values, and residual quantiles were evaluated. A dog-only variance model and leave-one-dog-out summaries assessed sensitivity. Session-specific bias, sample SD, MAE and RMSE were calculated. Post hoc strata of manual rate ≤25 and >25 breaths/min were descriptive only, because the upper stratum contained D03 exclusively. D03 was further examined using its minute-wise differences, camera/manual ratios and contribution to the total absolute and squared error. No observation was removed as an outlier. With four dogs, normality, constant variance and a rate effect independent of dog identity cannot be established reliably. No equivalence test was performed because no clinical equivalence margin had been specified. The agreement and waveform analyses used Python 3.12, NumPy 2.0.2 and SciPy 1.13.1. The supplementary FFT analysis used NumPy 2.3.5. Signal extraction and the initial time domain analysis used MATLAB.

## 3. Results

### 3.1. Respiratory Waveforms and Processing Verification

**Figure 4:**
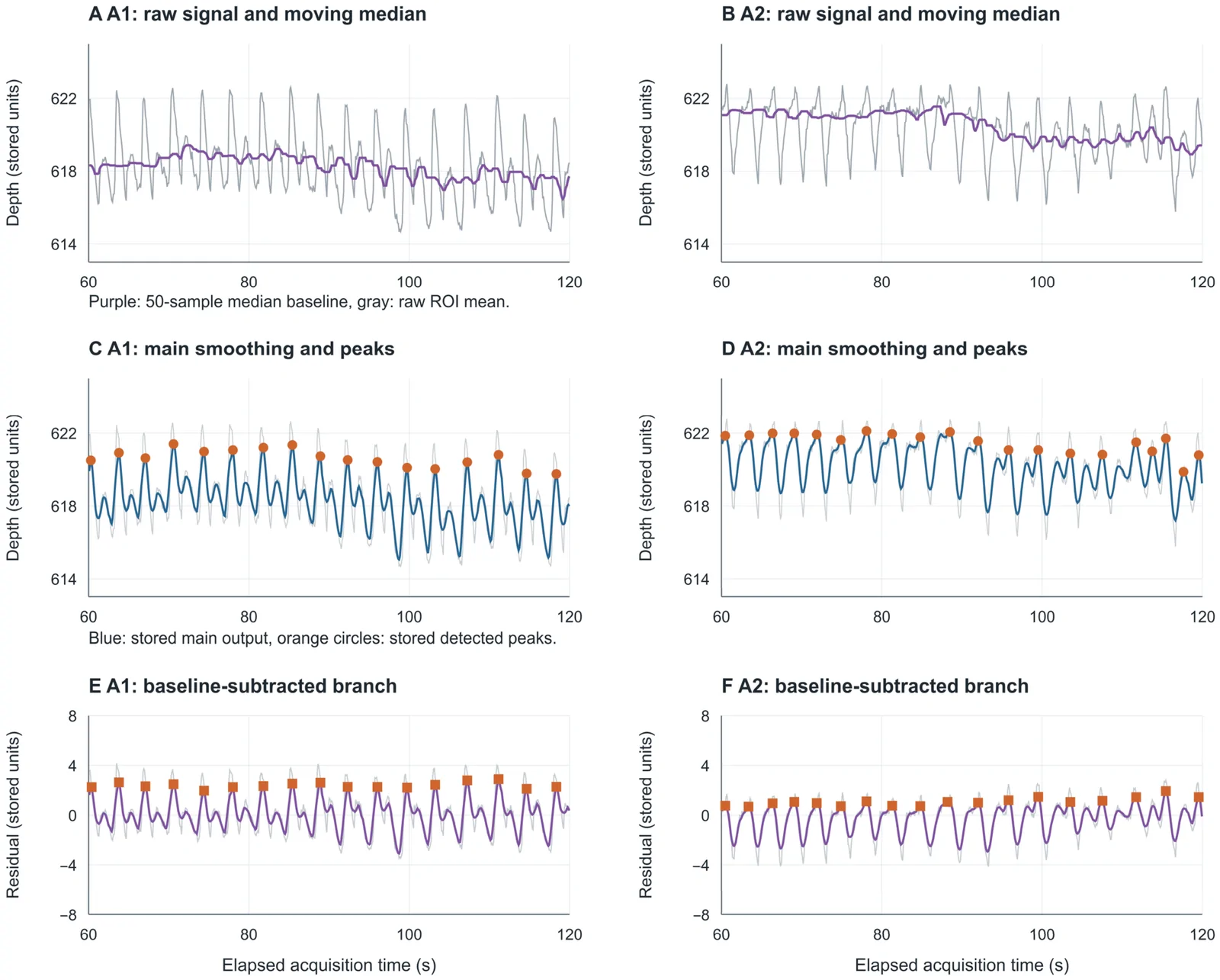
Signal processing stages for the common elapsed-time interval 60–120 s. (**A**,**B**): Raw ROI mean (gray) with the 50-sample median baseline (purple). (**C**,**D**): Raw signal, stored main smoothing (blue) and stored peaks (orange circles). (**E**,**F**): Baseline-subtracted residual (gray), its nine-sample moving average (purple) and experimental peaks (orange squares). Each column uses the same recorded input throughout. Experimental outputs illustrate retrospective processing and are not the clinical spectral estimates.

Recordings A1 and A2 contained 2964 and 2959 depth frames, respectively. Filename timestamps spanned 300.002 and 299.901 s. Both had a median interframe interval of 0.100 s, with maximum gaps of 0.202 and 0.402 s. Duration estimated from frame count at 10 frames/s was 296.4 and 295.9 s, respectively. Figure 3 and Figure 4 show the selected ROIs and corresponding recorded signals. Acquisition-relative time is shown with the first sample at zero. The original MATLAB time vector was shifted by 0.1 s for display only. The mean rates calculated as (N − 1)/timestamp span were 9.8766 and 9.8633 frames/s. Timestamp span, rather than N/10, defines the observed camera time base.

Mean depth signals ranged from 613.25 to 625.72 stored units in A1 and from 603.19 to 624.54 in A2. Recalculation of the nine-sample moving average reproduced the MATLAB output exactly, including the endpoint behavior. Peak detection reproduced 85 candidate peaks in A1 and 88 in A2 at the same timestamps, with median interpeak estimates of 16.41 and 18.73 breaths/min. This numerical consistency check verifies the time domain implementation. We did not acquire independent breath event annotations, so sensitivity and positive predictive value for individual breaths could not be determined.

Applying the experimental branch to the same recorded raw signals yielded 74 and 77 peaks, with median interpeak estimates of 15.76 and 16.19 breaths/min. Baseline subtraction reduced slow changes in depth while preserving oscillatory residuals in the displayed interval. Secondary deflections and varying cycle shape remained visible. Peak prominence and minimum separation therefore changed which candidate oscillations were retained, even with identical input signals (Figure 4).

**Figure 5:**
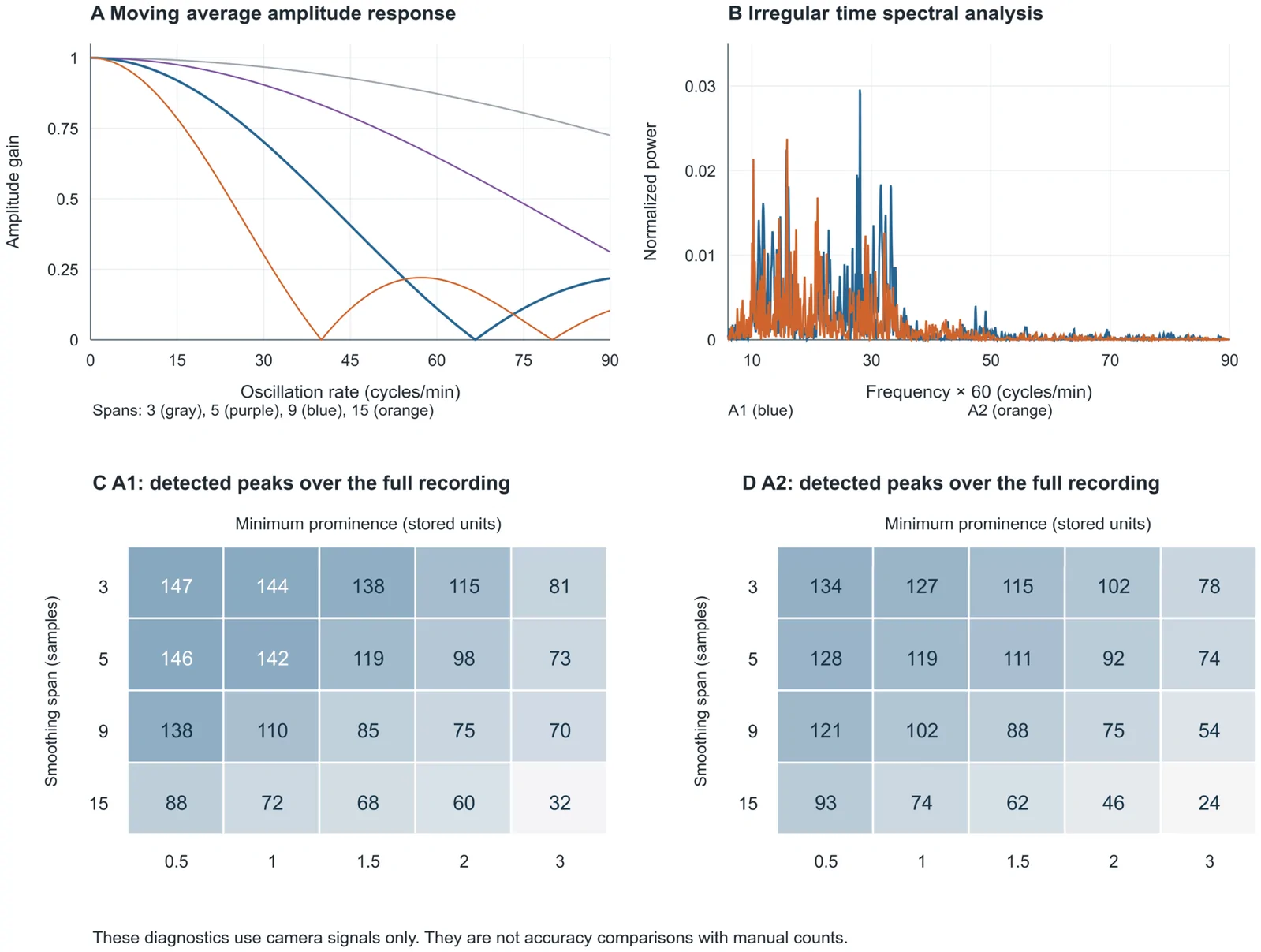
Processing diagnostics. (**A**): Theoretical rectangular moving average amplitude responses at 10 samples/s. (**B**): Normalized Lomb–Scargle periodograms of the actual mean-centered raw signals at their original timestamps. No interpolation was used. (**C**,**D**): Total candidate peaks across each complete recording for the indicated smoothing spans and prominence thresholds, without minimum separation. The main MATLAB settings correspond to span 9 and prominence 1.5. Candidate peak counts are not minute-level accuracy results.

The nominal nine-sample moving average had amplitude gains of 0.94, 0.86, 0.51 and 0.076 at 13, 20, 40 and 62 cycles/min, respectively, and its first zero at 66.67 cycles/min. Thus, substantial attenuation is expected near the upper observed breathing rate range even before applying a prominence threshold. Across the 20 exploratory parameter combinations, the total detected peaks ranged from 32 to 147 in A1 and from 24 to 134 in A2 (Figure 5). These counts quantify sensitivity to processing choices. They do not identify a validated optimal parameter set.

The largest raw signal periodogram peaks occurred at 28.08 cycles/min in A1 and 15.90 cycles/min in A2. Their difference from the corresponding median interpeak estimates shows that spectral dominance and detected cycle spacing are not interchangeable summaries of these nonstationary, nonsinusoidal traces. Time-resolved reference annotations would be needed to determine which waveform deflections represent individual breaths.

In the supplementary estimator comparison, FFT minute medians ranged from 15.45 to 33.60 breaths/min in A1 and from 17.70 to 28.65 breaths/min in A2. Corresponding median interpeak estimates ranged from 13.46 to 27.24 and from 15.36 to 21.79 breaths/min (Supplementary Table S6). In A1 minutes 2 and 3, FFT values of 33.60 and 31.80 were approximately twice the peak-based values of 16.64 and 16.19. These differences demonstrate estimator dependence. Without an event-level reference, they do not identify the physiological fundamental. We retained all 60 short windows, including one A2 window at the lower search boundary of six breaths/min.

The new ROI analysis produced finite signals for all 27 configurations in each recording. The minimum valid-pixel fraction across all frames and ROIs was 98.48% in A1 and 98.26% in A2. Across the 26 nonbaseline ROIs, full-record waveform correlations with the baseline ranged from 0.914 to 0.999 in A1 and 0.884 to 0.999 in A2. Nevertheless, the largest absolute changes in minute-level FFT estimates were 7.50 and 7.80 breaths/min, respectively. The largest 10 s changes were 17.70 and 30.00 breaths/min. In the latter A2 comparison, estimates of 36.0 and 6.0 breaths/min occurred despite a within-window waveform correlation of 0.996. Inspection of the corresponding spectra shows a change in which competing component had the greatest magnitude. The 6.0 breaths/min estimate lies at the lower search boundary. These outcomes demonstrate spatial sensitivity of the supplementary estimator, not accuracy against independent breaths (Supplementary Materials S2, Tables S1 and S2 and Figures S2 and S3).

### 3.2. Available Paired Measurements

Four dogs contributed ten complete pairs each, totaling eight sessions and 40 min level pairs. No pair was missing within this analyzed 4 × 2 × 5 subset. The wider table also contained ten manual-only D04 positions and ten camera-only D06 positions, which did not enter agreement calculations (Supplementary Table S8). All 40 included manual entries agreed with the photographed tally sheets. Manual counts ranged from 13 to 62 breaths/min and ToF values from 13 to 72 breaths/min. Pooled means were 24.80 and 27.13 breaths/min.

### 3.3. Agreement with Concurrent Manual Counting

Mean bias was +2.33 breaths/min (95% CI −3.60 to +8.25), MAE 3.93 breaths/min and RMSE 6.64 breaths/min. The median absolute difference was 1.50 breaths/min, and signed differences ranged from −8 to +21. Twenty-five pairs (62.5%) were within two breaths/min and 31 (77.5%) within five breaths/min. These percentages are descriptive repeated observations, without a claim that the selected tolerances define clinical acceptability.

Dog-specific errors are shown in Table 2. D03 had a bias of +7.90 and MAE of 10.90 breaths/min, compared with MAEs of 0.60, 2.80 and 1.40 in D01, D02 and D05. The standard deviation of differences was 10.42 breaths/min in D03, versus 0.92, 3.53 and 1.96 in the other dogs. This pronounced heterogeneity limits a single common variance description.

Positive differences indicate higher ToF estimates. Each dog contributed two sessions of five paired minutes. Pooled MAE and RMSE are descriptive. The independent sample comprises four dogs.

**Figure 6:**
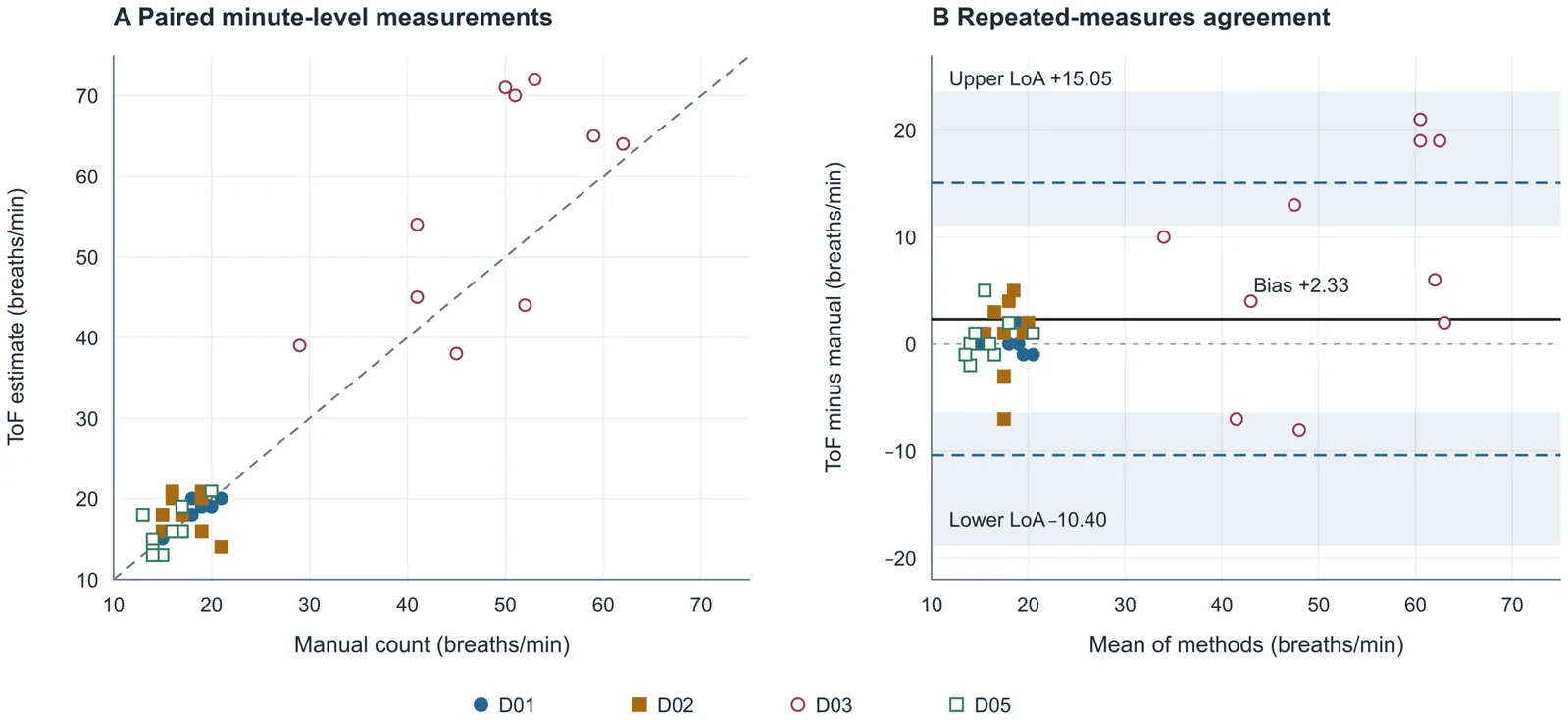
Agreement of ToF estimates with concurrent manual counting. (**A**): Paired values and the identity line. (**B**): Difference against the mean of methods. Solid black line indicates bias, blue dashed lines indicate repeated-measures limits of agreement, and shaded bands indicate their separate model-based 95% confidence intervals. Symbols identify dogs and some repeated observations overlap. The limits assume common error dispersion despite substantial dog-specific heterogeneity.

The nested model estimated variance components of 5.99 for dogs, 10.62 for sessions within dogs and 25.53 for residual variation, in squared breaths/min. The total standard deviation of differences was 6.49 breaths/min. Repeated-measures limits of agreement were −10.40 and +15.05 breaths/min (Figure 6). The corresponding hierarchical agreement estimates and their 95% confidence intervals are summarized in Table 3. Model-based 95% bootstrap-t confidence intervals were −18.91 to −6.38 for the lower limit and +11.06 to +23.60 for the upper limit. These are exploratory model summaries. The small number of dogs and unequal error dispersion prevent a firm assertion of population coverage. The conditional residual and quantile plots show heterogeneous dispersion and tail departures (Supplementary Section S4, Figure S1), supporting cautious interpretation of the common variance model.

**Table 3:** Hierarchical agreement estimates and uncertainty.

Quantity	Estimate	95% Confidence Interval
Mean bias (breaths/min)	+2.33	−3.60 to +8.25
Lower limit of agreement (breaths/min)	−10.40	−18.91 to −6.38
Upper limit of agreement (breaths/min)	+15.05	+11.06 to +23.60
Between-dog variance	5.99	Not separately estimated
Session-within-dog variance	10.62	Not separately estimated
Residual variance	25.53	Not separately estimated
Total standard deviation (breaths/min)	6.49	Not separately estimated

Bias CI: t interval using four dog means (3 degrees of freedom). Limit CIs: 50,000 parametric bootstrap-t replicates of the nested model. Variances are in (breaths/min)^2^. Confidence intervals for variance components were not calculated. All paired observations were retained.

**Figure 7:**
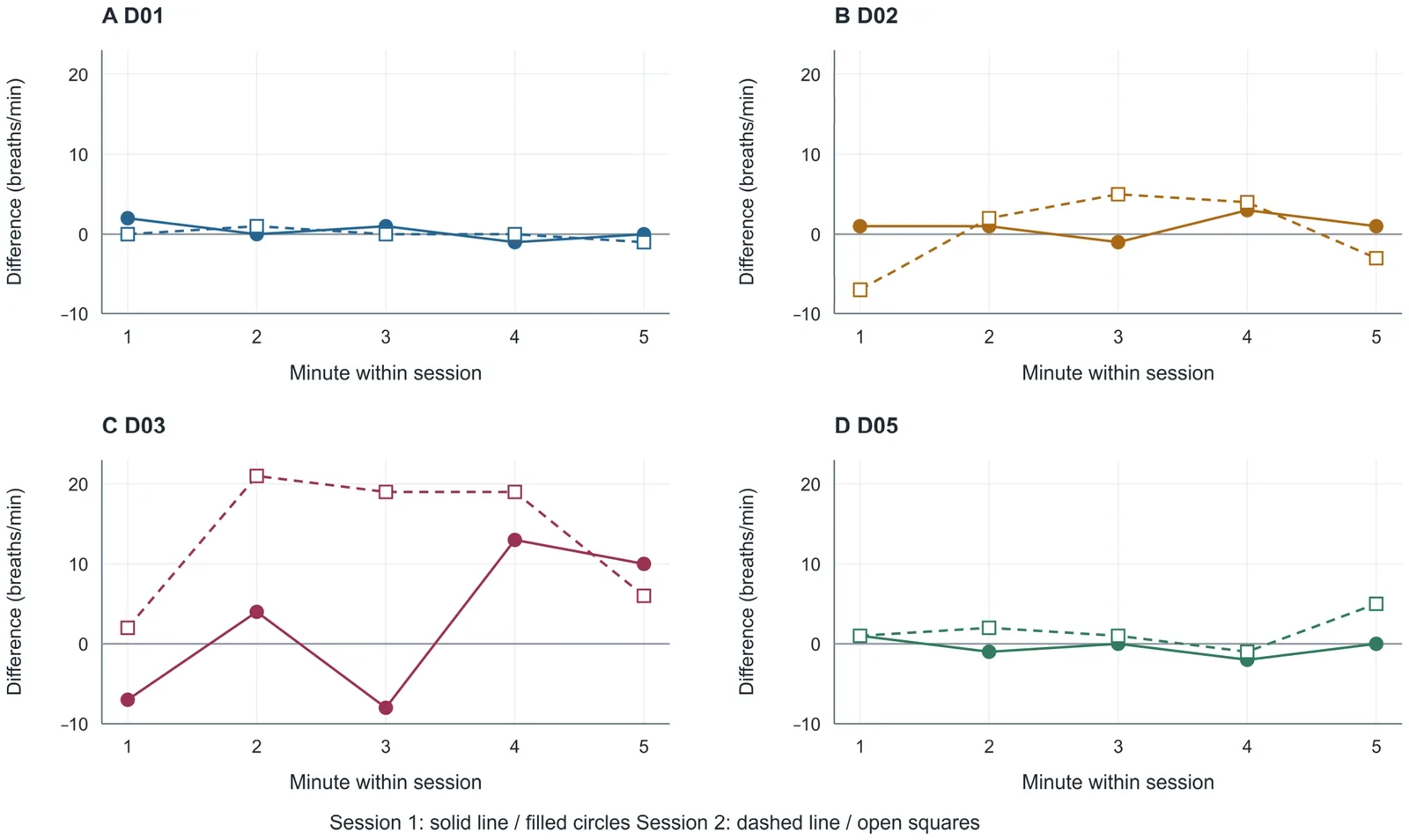
ToF-minus-manual differences across the five minutes of each session, shown separately for all four dogs using the same vertical scale. Filled circles and solid lines identify session 1. Open squares and dashed lines identify session 2. Lines connect sequential minute summaries rather than continuous respiratory waveforms. The sustained positive differences in the second D03 session demonstrate why pooled errors require dog- and session-level interpretation.

Session-specific errors are reported in Table 4 and Figure 7. D03 had mean differences of +2.40 and +13.40 breaths/min in sessions 1 and 2, respectively. In session 2, the five differences were +2, +21, +19, +19 and +6 breaths/min, showing a sustained middle-session deviation rather than a single isolated outlier. D03 accounted for 69.4% of the summed absolute error and 90.8% of the summed squared error. Its ten camera/manual ratios ranged from 0.84 to 1.42, without persistent doubling or halving (Supplementary Table S7). These minute summaries cannot exclude transient harmonic selection.

**Table 4:** Session-specific errors in breaths/min. Each session contains five paired observations. SD uses denominator n − 1.

Dog	Session	n	Bias	SD	MAE	RMSE
D01	1	5	+0.40	1.14	0.80	1.10
D01	2	5	+0.00	0.71	0.40	0.63
D02	1	5	+1.00	1.41	1.40	1.61
D02	2	5	+0.20	5.07	4.20	4.54
D03	1	5	+2.40	9.61	8.40	8.92
D03	2	5	+13.40	8.73	13.40	15.51
D05	1	5	−0.40	1.14	0.80	1.10
D05	2	5	+1.60	2.19	2.00	2.53

The post hoc stratification by manual respiratory rate is summarized in Table 5. The >25 breaths/min stratum consists entirely of D03, while the other three dogs contribute only to the ≤25 stratum. The lower-stratum MAE of 1.60 and upper-stratum MAE of 10.90 therefore cannot be interpreted as an independent rate effect. A dog-only agreement model yielded the same point limits in this balanced dataset, with a different variance partition. Leave-one-dog-out MAEs ranged from 1.60 to 5.03 breaths/min and RMSEs from 2.32 to 7.65. D03 remains in the primary analysis.

**Table 5:** Post hoc strata defined by manual respiratory rate. Rates and errors are in breaths/min.

Manual Rate	Dogs	n	Bias	SD	MAE	RMSE
≤25	3	30	+0.47	2.32	1.60	2.32
>25	1	10	+7.90	10.42	10.90	12.65

## 4. Discussion

Our results demonstrate the feasibility of extracting canine respiratory motion signals from regional ToF depth measurements. The waveform analyses characterize how smoothing, peak criteria and spectral estimation affect the resulting summaries. Agreement with manual counting varied substantially between dogs and sessions. The comparison of FFT and peak-based estimates on identical signals provides a direct assessment of processing dependence, while the paired clinical analysis describes the performance observed during routine measurements.

The measured quantity is a change in surface distance. It reflects the visible surface, including fur, and is influenced by camera orientation, posture and ROI placement. Spatial averaging can attenuate independent pixel noise, but it also combines regions that may move with different amplitudes or phases. A fixed ROI can include a different anatomical surface after movement, while frame-dependent rejection of depth values can change the pixels contributing to the mean. A stable average depth oscillation is consequently a surrogate for respiratory motion rather than a direct measurement of lung volume or airflow. Respiratory effort and airflow can diverge, so neither a visible oscillation nor its absence alone establishes a particular apnea mechanism. The anatomical ROI comparison by Kojima et al. supports evaluating regional waveform fidelity explicitly [23].

The ROI perturbation experiment shows that strong waveform similarity does not guarantee stable dominant frequency estimates. Small spatial changes altered the relative magnitudes of competing spectral components in the illustrated windows. Minute medians reduced some short-window variation but still differed by up to 7.80 breaths/min from the fixed baseline. The new rectangles cover a limited neighborhood of one visible trunk region in two recordings from one dog. These findings do not establish robustness across anatomical regions, operators, postures or animals. They identify a spatial sensitivity that should be addressed when defining a fixed algorithm for independent validation.

Temporal processing must be matched to the range of respiratory patterns being studied. Smoothing suppresses high-frequency fluctuations, but a near-one-second averaging window can also attenuate faster respiratory oscillations. A peak prominence threshold then interacts with signal amplitude and smoothing. The experimental minimum separation of 2.5 s imposes an additional constraint that is incompatible with retaining every breath at sustained rates substantially above 24 breaths/min. The observed peak count sensitivity and the calculated frequency response quantify these mechanisms. These findings concern the peak-based MATLAB branch. They cannot be assigned as the cause of disagreement in the clinical comparison, which used spectral estimates.

Spectral estimation summarizes periodic structure within an observation window. Breathing, harmonics and body motion can contribute competing components. In our supplementary FFT analysis, the 10 s duration gives an unpadded bin spacing of 0.1 Hz, equivalent to six breaths/min. Zero padding provides a denser grid but retains the resolving limits of the finite window [25]. The approximately twofold differences between FFT and peak estimates in parts of A1 illustrate this ambiguity. We used Lomb–Scargle analysis as a complementary description of spectral content at the original timestamps [27]. A1 and A2 are D05 recordings, so their spectral behavior cannot establish the cause of D03 deviations.

D03 accounted for most of the error, with sustained overestimation in its second session. We examined its minute-wise differences and camera/manual ratios to test whether a persistent harmonic multiplier could explain the result. Ratios of 0.84–1.42 do not show consistent doubling or halving. Nearest integer rounding cannot explain differences of +19 to +21 breaths/min. We also quantified the frequency axis offset in our two development FFT implementations. Their spacing of f_s_/(NFFT − 1), rather than f_s_/NFFT, produces an offset of approximately +0.15 to +0.20 breaths/min at 10 Hz and NFFT = 2000 over 0.1–1.5 Hz. Even if present in the clinical calculation, this offset would be too small to explain the D03 deviations. The supplementary implementation uses exact FFT bin frequencies. We did not retain D03 window spectra, selected bins and synchronized breath event annotations. Transient harmonic selection, motion, manual undercounting and interval mismatch therefore remain possible contributors rather than established causes.

Time alignment remains a possible contributor whose magnitude cannot be estimated retrospectively. The timestamp spans and interframe gaps show why nominal frame count should not define interval boundaries. Prospective verbal coordination, a software start lead time and a synchronization signal do not quantify residual alignment. In particular, a 5 s software start lead is not proof of a 5 s misalignment in the analyzed pairs. Accurate comparisons require a retained mapping from camera timestamps to reference start and end markers, explicit aggregation of short spectral windows, and duration normalization of partial counting intervals.

Concurrent manual counting is clinically relevant but is an operational reference with uncertainty. Camera estimates were concealed from the counting observer, reducing the direct influence of the displayed result. However, neither repeat counts by independent observers nor breath event annotations were available. Shallow movements, irregular breathing and cycles at interval boundaries may be difficult to classify, especially at higher rates. Available monitor entries did not provide an independently verified, synchronized respiratory reference for these intervals. Future validation should quantify reference repeatability and retain an instrumental respiratory signal where feasible [29].

The canine literature provides a useful caution against interpreting a favorable aggregate statistic as interchangeable measurements. In 19 hospitalized dogs, Warhoe et al. found that a wireless harness could correlate strongly with manual measurements while individual differences exceeded their predefined clinical thresholds. Coat length and chest conformation also influenced performance [5]. This does not demonstrate the same effects in ToF sensing, but supports evaluating canine morphology explicitly. The inertial, radar and thermal animal studies address different observation conditions and cannot supply an accuracy claim for this depth camera implementation [4,7,8].

The clinical literature supports three priorities for further validation: an independently recorded respiratory reference, systematic changes in acquisition conditions, and a defined output availability denominator. Addison et al. evaluated all three in healthy adults [20].

L’Her et al. extended noncontact surface imaging to 44 adult ICU patients, including mechanically ventilated and high-flow nasal oxygen groups [34]. Those settings and algorithms differ from the canine study, so their errors cannot serve as acceptance thresholds for this implementation.

We compared temporal estimators on the same A1/A2 ROI signals to examine an algorithmic component directly within our dataset. Reproducing complete published pipelines would also require their spatial extraction, body region localization or additional sensing modalities [13,17,22,23,24]. Our scalar ROI signals do not contain the information needed to reproduce those upstream operations. A valid comparison of complete algorithms would require standardized image-level inputs and synchronized reference signals, followed by independent evaluation in dogs. The present comparison therefore addresses spectral versus peak-based rate estimation.

Continuous clinical utility requires both useful estimates and sufficient availability. Jorge et al. reported respiratory rate availability for 63.1% of the time with a valid reference during extended video monitoring of 15 postoperative ICU patients [35]. Our 40 complete pairs out of 60 table positions describe completeness of the retained results table, not continuous availability or acquisition success. The absence of a complete attempt and rejection log prevents a comparable availability calculation.

Finally, respiratory rate agreement should be distinguished from respiratory event classification. Schätz et al. investigated depth-derived signals alongside a separately developed classifier using polysomnographic data [36]. A count of maxima in the present ROI signal does not implement such a classifier. Likewise, prior studies of regional chest motion and respiratory effort do not convert the present scalar depth average into a validated airflow or volume measurement [14,15,16].

Several limitations constrain inference. Only four independent dogs contributed paired values, and the highest rates occurred in one dog. Repeated minutes cannot separate animal identity from rate effects. The normal error, common variance model is approximate, with residual heterogeneity and tail departures. Serial dependence beyond the session effect was not modeled, and 46.9% of bootstrap refits reached a zero variance component. The additional ROI analysis assessed only predefined geometric perturbations in two D05 recordings. Independent operator selections and the original session-specific masks were unavailable. Session-specific clinical FFT configurations and residual synchronization were incompletely documented. Our additional development image series lacked linked respiratory references and were excluded from clinical validation. Continuous unattended monitoring, alarm latency and clinical outcomes were not evaluated.

Breed type, body mass, age, sex and coat length are now reported for the four dogs, but body condition scores, quantitative thoracic conformation measures and standardized clinical condition categories were unavailable. The number of rejected recording attempts was not logged, and camera height, pitch angle, ambient illumination, infrared interference and image occupancy were not measured. These omissions limit reproducibility of the acquisition geometry and assessment of selection effects.

A subsequent validation should freeze the acquisition settings and algorithm before testing on independent dogs, preserve the selected ROIs and all exclusion decisions, and retain synchronized reference intervals. It should include enough animals across body sizes, coat characteristics, postures and respiratory rates to separate these sources of variation. Predefined acceptable errors and a signal availability measure should accompany repeated-measures agreement analysis in that independent validation [30].

## 5. Conclusions

ToF depth imaging yielded oscillatory surface distance signals in the studied dogs. The supplementary analyses demonstrated the dependence of frequency estimates on both temporal processing and ROI geometry. A fully specified image-to-estimate implementation is provided for the additional ROI analysis. The findings support the technical feasibility of signal extraction while identifying unresolved limitations in respiratory rate estimation.

The clinical comparison comprised 40 paired minutes from four dogs, with exploratory repeated-measures limits of agreement of −10.40 to +15.05 breaths/min. Errors were concentrated in one dog and particularly one session.

The method has not been validated as a reliable respiratory rate monitor across dogs or respiratory rate ranges. Further validation requires more independent animals, fixed processing configurations, reproducible ROI selection and synchronized respiratory references.

## Figures and Tables

**Table 1:** Numerical settings and their roles in the MATLAB implementation.

Parameter	Main Workflow	Experimental Branch
Nominal frame rate	10 frames/s	Inherited from main script
ROI	Fixed four-vertex polygon or full frame	Same extracted ROI mean
Depth validity range	1 to 2000 inclusive	Inherited from main script
Baseline removal	None	Moving median, 50 samples
Smoothing	smooth(s,10)	smooth(r,10)
Minimum prominence	1.5 depth units	2 depth units
Minimum peak separation	Not specified	2.5 s
Peak search start	Full processed vector	Sample 7
Rate summary	60/median interpeak interval	Separate time bin histogram

**Table 2:** Errors relative to concurrent manual counting, in breaths/min.

Dog ID	Pairs	Manual Range	Mean Difference	MAE	RMSE
D01	10	15 to 21	+0.20	0.60	0.89
D02	10	15 to 21	+0.60	2.80	3.41
D03	10	29 to 62	+7.90	10.90	12.65
D05	10	13 to 20	+0.60	1.40	1.95
Pooled	40	13 to 62	+2.33	3.93	6.64

## Data Availability

Data and code supporting the findings of this study are available from the corresponding author upon reasonable request.

## Supplementary Materials

The following supporting information can be downloaded at: https://www.mdpi.com/article/10.3390/s26216986/s1, Supplementary Materials S1 contains the statistical derivations, waveform analyses, spectral implementations and clinical data summaries. Supplementary Materials S2 reports the additional ROI sensitivity experiment, complete parameters, Tables S1 and S2 and Figures S2 and S3. Data S1 and Code S1 contain the clinical pairs and executable analyses. Data S2 and Code S2 contain the ROI configuration, frame manifest, extracted signals, all window and minute outputs, and executable image-to-estimate processing. The corresponding depth frames are supplied separately in Raw Depth Data S2. Figures S2 and S3 are provided at 1200 dpi and in vector formats.
