# Supplementary material for: Technical Feasibility of Respiratory Monitoring in Dogs Using Time-of-Flight Depth Imaging
Source: Sensors (Basel). 2026 Nov 5;26(21):6986. doi: 10.3390/s26216986 (PMC13766340; doi:10.3390/s26216986)
Supplement: Supplementary file 1 [file sensors-26-06986-s001.zip › sensors-4627966-Supplementary Materials S1.pdf]

Supplementary Materials S1

Supplementary Materials S1 for Technical Feasibility of Respiratory Monitoring in Dogs Using Time of Flight Depth Imaging. We report the agreement calculations, model diagnostics and numerical waveform reconstruction.

S1 Paired data and independent observations

We analyzed 40 minute-level pairs from four dogs, with two sessions and five paired minute summaries per session. Manual values were checked against the photographed tally sheets from the March 2022 recording sessions. The records did not contain independently annotated breath-event timestamps.

The signed difference is camera minus manual. Exact pooled totals are $\Sigma d = 93$, $\Sigma|d| = 157$ and $\Sigma d^2 = 1763$ across 40 pairs. Thus bias = 2.325, MAE = 3.925 and RMSE = $\sqrt{(1763/40)} = 6.6389005$ breaths/min. MAE, RMSE and tolerance percentages summarize this sample. They do not imply 40 independent patients.

Table S1. Bias and within-dog dispersion, in breaths/min. Each session mean contains five differences. Each dog SD uses ten differences and denominator 9.

Dog	Session 1 bias	Session 2 bias	Dog mean	SD of differences
D01	+0.40	+0.00	+0.20	0.92
D02	+1.00	+0.20	+0.60	3.53
D03	+2.40	+13.40	+7.90	10.42
D05	-0.40	+1.60	+0.60	1.96

S2 Nested agreement model and exact point estimates

The model was $d(i,j,k) = \mu + u(i) + v(i,j) + e(i,j,k)$, with independent normal dog, session-within-dog and residual effects. For a balanced design with $g = 4$ dogs, $b = 2$ sessions and $m = 5$ minutes, let MS_D, MS_S and MS_E denote the nested ANOVA mean squares. Their degrees of freedom are 3, 4 and 32, respectively. The total sum of squared deviations is $3\ MS\_D + 4\ MS\_S + 32\ MS\_E = 1546.775$.

Table S2. Mean squares and their expectations in the balanced nested model.

Mean square	Observed value	Expected value
Dog	138.49166667	$\sigma^2E + 5\sigma^2S + 10\sigma^2D$
Session within dog	78.62500000	$\sigma^2E + 5\sigma^2S$
Residual	25.52500000	σ^2E

The component estimates are $\sigma^2E = MS\_E = 25.525$, $\sigma^2S = (MS\_S - MS\_E)/5 = 10.620$, and $\sigma^2D = (MS\_D - MS\_S)/10 = 5.9866667$. All are positive. In this balanced design they equal the interior restricted

maximum-likelihood (REML) estimates. An independent numerical REML fit using the full 40 × 40 covariance matrix reproduced all three estimates within 0.00001.

For a new paired minute from a new dog/session, the estimated total variance is $V = \sigma^2_D + \sigma^2_S + \sigma^2_E = 42.1316667$. Equivalently, V = 0.1 MS_D + 0.1 MS_S + 0.8 MS_E. The total SD is 6.4908911 breaths/min and the exploratory 95% limits of agreement are 2.325 ± 1.96 × 6.4908911 = -10.3971465 to +15.0471465 breaths/min. Repeated-measures and hierarchical agreement principles are described by Bland and Altman and Parker et al. [S1,S2].

S3 Confidence intervals and bootstrap implementation

For mean bias, the independent observations are the four dog means: 0.2, 0.6, 7.9 and 0.6 breaths/min. The standard error is their sample SD divided by √4, equivalently √(MS_D/40). The 95% t interval uses 3 degrees of freedom and t(0.975,3) = 3.1824463, giving -3.5966524 to +8.2466524 breaths/min. Normality of the population of dog means cannot be established with four dogs.

For each limit, a parametric bootstrap-t extension of hierarchical agreement methods was used [S2,S3]. In each of 50,000 replicates (NumPy generator seed 20260911), four dog effects, eight nested session effects and 40 residuals were simulated from independent normal distributions with the fitted component variances. The same 4 × 2 × 5 data structure was retained and the model was refitted.

Nonnegative variance components were enforced by ordering the three expected mean squares $\lambda_D \geq \lambda_S \geq \lambda_E$. When adjacent estimated mean squares violated that order, they were pooled using their degrees of freedom as weights, iterating until all constraints held. This is constrained REML in the orthogonal balanced nested model. Boundary solutions were retained, not discarded. At least one component was zero in 46.878% of replicates.

For an interior fit, the delta-method standard-error calculation uses $\mathrm{Var}(\hat{V}) \approx 2[(0.1\ MS\_D)^2/3 + (0.1\ MS\_S)^2/4 + (0.8\ MS\_E)^2/32]$, $\mathrm{Var}(\hat{\mu}) = MS\_D/40$, $\mathrm{Var}(\sqrt{\hat{V}}) \approx \mathrm{Var}(\hat{V})/(4\hat{V})$, and $\mathrm{SE(LoA)} \approx \sqrt{[\mathrm{Var}(\hat{\mu}) + 1.96^2\ \mathrm{Var}(\sqrt{\hat{V}})]}$. The factor in the last denominator is the variance $\hat{V}$, not its square root. The observed SE for either limit is 2.7705024 breaths/min. When mean squares are pooled at a boundary, the corresponding coefficients and degrees of freedom are summed before applying the variance formula. This boundary standard error is an approximation.

For each bound L, the bootstrap pivot is $z^* = (L^* - \hat{L})/\mathrm{SE}(L^*)$. Using its empirical 2.5th and 97.5th percentiles, the interval is $[\hat{L} - q(0.975)\mathrm{SE}(\hat{L}), \hat{L} - q(0.025)\mathrm{SE}(\hat{L})]$. The resulting 95% intervals are -18.9108 to -6.3794 for the lower limit and +11.0557 to +23.5992 for the upper limit. These are separate model-based intervals, not a simultaneous confidence band or a guarantee of 95% empirical coverage in dogs. They do not supply clinical acceptance thresholds.

S4 Model diagnostics and sensitivity

Conditional fitted differences were calculated using the random-intercept predictions. The residual plot and quantile plot show heterogeneous dispersion and tail departures (Figure S1). Repeated minutes are correlated within a dog/session, and residual serial dependence beyond that model was not estimated. Four dogs are insufficient to verify the assumed normal dog-effect distribution or to fit a credible rate-by-dog interaction. The model is therefore a transparent exploratory approximation, not a definitive clinical coverage model.

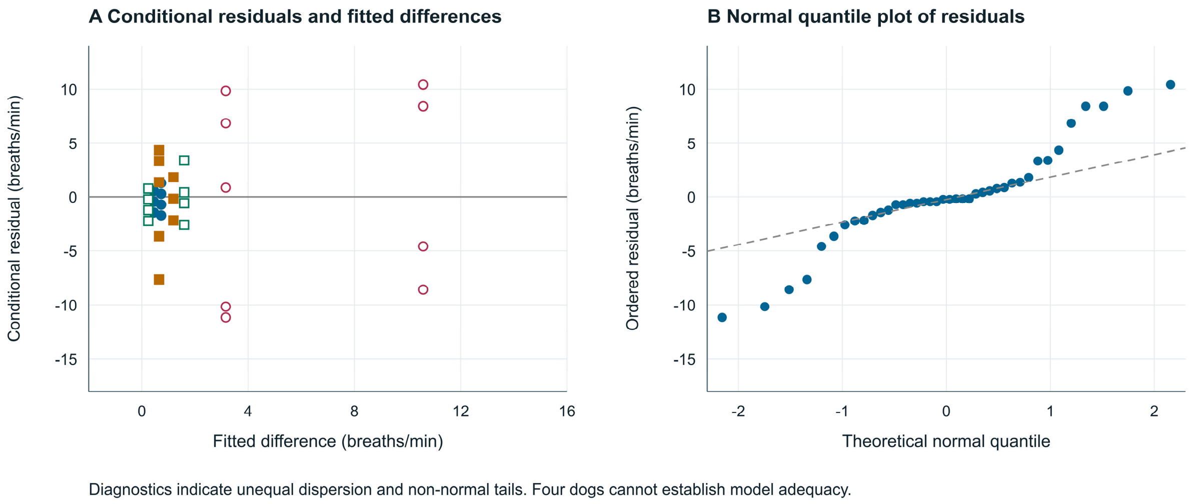

Figure S1. Conditional model diagnostics. A: residuals against fitted differences, colored by dog as in the main manuscript. B: ordered residuals versus normal quantiles. The dashed line uses the residual quartiles. These checks reveal departures rather than proving model adequacy.

A dog-only model produced the same total variance and point limits for this balanced dataset. Its within-dog mean square was 31.425 and its between-dog variance was 10.7066667. Their sum again equals 42.1316667. Equal point limits do not mean session effects are absent. The nested model explains that substantial variability occurs between the two sessions in D03.

Table S3. Leave-one-dog-out sensitivity, in breaths/min. The primary result retains all four dogs. No subgroup was chosen as a replacement primary analysis.

Dog omitted	Remaining pairs	Bias	MAE	RMSE
D01	30	3.033	5.033	7.649
D02	30	2.900	4.300	7.409
D03	30	0.467	1.600	2.324
D05	30	2.900	4.767	7.583

S5 Waveform analysis and reconstruction

The paired analysis used minute-level rates obtained by FFT and spectral interpolation. For the supplementary waveform analyses, we extracted timestamps, raw ROI means, smoothed means and peak coordinates. Their lengths

matched the corresponding TIFF sequences. We used the saved ROI signals rather than redefining polygons because exact vertex coordinates had not been saved separately. The two manual sequences in the D05 session were 14, 17, 16, 15, 14 and 20, 17, 14, 14, 13 breaths/min. A timestamp mapping linking these manual sequences to independently annotated breath events was not available, so they could not be used for event-level validation.

A symmetric nine-sample mean reproduced every smoothed value, using endpoint spans of 1, 3, 5 and 7 samples. Peak detection at 1.5 reproduced 85 in A1 and 88 in A2 at the same raw inputs, a zero-padded 50-sample median with samples k-25 to k+24, the nine-sample mean, prominence 2 and a 2.5-s separation, beginning at sample 7. It yielded 74 and 77 peaks. The original development figures used different ROI traces, so their outputs were not acquired.

Table S4. Technical summaries. Rates are in breaths/min. Peak counts cover the full recording and are not directly comparable with an independently timed one-minute manual count.

Quantity	A1	A2
Samples	2964	2959
Timestamp span (s)	300.002	299.901
Main-branch peaks	85	88
Main median-interval RR	16.4136	18.7266
Experimental-branch peaks	74	77
Experimental median-interval RR	15.7646	16.1943
Parameter-grid count range	32–147	24–134

The same elapsed 60–120-s interval is shown for both recordings. Normalized Lomb–Scargle spectra used original timestamps and the raw signal after mean subtraction, over 0.10–1.50 Hz at 0.001-Hz steps [S4]. Power values are dimensionless and were not interpreted as a validated signal-to-noise ratio. No significance or false-alarm threshold was applied. The filter response is an algebraic diagnostic under uniform 10-Hz sampling and does not model the nonlinear median filter.

S6 Spectral implementations and comparison of estimators

We developed two FFT implementations for extracting respiratory rate from periodic changes in mean ROI depth. Both subtract the segment mean, apply a Hann window and use zero-padding to refine the frequency grid. Their segment definitions and treatment of timestamps differ, as summarized in Table S5. The first specifies 50 samples, which correspond to 10 s at 5 Hz or 5 s at 10 Hz. The second specifies a duration of 10 s. Our session records did not retain the processing configuration and short-window aggregation for each clinical minute. The table therefore describes our development implementations, while the fully specified supplementary comparison below defines the analysis performed on A1 and A2.

Table S5. Our development FFT implementations. Session-specific configuration records do not establish which complete configuration generated each clinical minute estimate.

Component	Variant A	Variant B
Window and step	50 samples each	10 s each, configured at 5 Hz
ROI	Central image region and motion checks	Model-based region and quality checks
Time resampling	No explicit resampling in implemented FFT path	Linear interpolation onto a uniform grid
Preprocessing	Mean subtraction and Hann window	Mean subtraction and Hann window, additional mean subtraction inside frequency routine
Spectral grid	Zero-padding to int(fs/0.005)	Zero-padding to int(fs/0.005)
Frequency selection	Largest detected local positive-spectrum magnitude peak	Largest detected local positive-spectrum magnitude peak
Output	Rounded frequency × 60, integer output	Rounded frequency × 60
Pre-FFT moving average	No nine-sample smoothing in implemented path	No nine-sample smoothing in implemented path
Minute aggregation	Not established for clinical table	Not established for clinical table

In these development routines, we constructed the frequency axis with linspace(-fs/2,fs/2,NFFT) and paired its positive half with the FFT bins starting at index zero. This gives a nonzero initial frequency and spacing fs/(NFFT - 1). We quantified the resulting offset as +0.153 to +0.195 breaths/min over 0.1-1.5 Hz at fs = 10 Hz and NFFT = 2000. This calculation characterizes the implementation error. Its contribution to the original clinical estimates cannot be assigned without session-specific configuration records. For the supplementary analysis, we used the exact FFT-bin frequencies k × fs/NFFT.

For the supplementary spectral analysis, we linearly interpolated A1/A2 raw ROI means at 3000 acquisition-relative time points from 0.0 to 299.9 s without extrapolation. Each nonoverlapping 100-sample window was mean-centered and multiplied by $w[n] = 0.5 - 0.5 \cos(2\pi n/99)$, n = 0,...,99. A 2000-point real FFT supplied magnitudes at $f[k] = k \times 10/2000$ Hz. We selected the maximum in the inclusive 0.10-1.50-Hz band. No moving-average smoothing, band-pass filter, quality rejection or further peak interpolation was used. Each minute value is the median of six unrounded frequency × 60 estimates. Display values alone are rounded to two decimals. Windowing and finite-duration spectral resolution are discussed by Harris [S5].

We compared these spectral estimates with 60 divided by the median interval between consecutive main-branch peaks within the same acquisition-relative minute [60m,60(m+1)), m = 0,...,4. Peak counts are reported separately because counts and median-interpeak estimates are different summaries. All 30 spectral windows per recording were retained. We used this explicitly parameterized comparison to evaluate estimator behavior without optimizing against the manual reference.

Table S6. Supplementary comparison of spectral and peak-based estimates on identical A1/A2 signals. Rates are in breaths/min. Each FFT median summarizes six 10-s windows.

Recording	Minute	FFT median	FFT window range	Peak count	Interpeak rate
A1	1	28.50	25.20-40.50	22	27.24
A1	2	33.60	30.30-36.00	17	16.64
A1	3	31.80	15.30-33.30	17	16.19
A1	4	15.45	12.30-24.30	14	13.62
A1	5	21.15	13.50-27.00	15	13.46
A2	1	20.55	6.00-38.40	20	20.66
A2	2	19.95	16.50-31.20	20	18.73
A2	3	28.65	20.10-30.60	19	21.79
A2	4	21.90	12.30-32.40	14	15.77
A2	5	17.70	6.30-21.90	15	15.36

A1 minutes 2 and 3 show approximate twofold differences between the spectral and interpeak summaries. These differences do not establish which estimator reflects the true respiratory rate. One A2 short window reaches the lower search boundary of 6 breaths/min and is retained in the analysis. All window-level values and the boundary flag are available from the corresponding author upon reasonable request. These outputs have no independent breath-event reference and are not used to explain the clinical D03 errors.

S7 D03 error concentration and causal checks

All original D03 pairs are retained in Table S7. The dog contributes 109 of the 157 summed absolute errors (69.43%) and 1601 of the 1763 summed squared errors (90.81%). Its second session contains sustained positive deviations in minutes 2-4. Nearest-integer rounding alone has a maximum absolute contribution of 0.5 breaths/min per camera estimate and cannot explain these deviations. Ratios of the minute estimates provide a check for a persistent harmonic multiplier but cannot rule out brief within-window spectral errors.

Table S7. Original D03 minute pairs. Differences are camera minus manual. Rates and differences are in breaths/min.

Session	Minute	Manual	Camera	Difference	Ratio
1	1	45	38	-7	0.84
1	2	41	45	+4	1.10
1	3	52	44	-8	0.85
1	4	41	54	+13	1.32
1	5	29	39	+10	1.34
2	1	62	64	+2	1.03
2	2	50	71	+21	1.42
2	3	51	70	+19	1.37
2	4	53	72	+19	1.36
2	5	59	65	+6	1.10

We retained D03 waveform images but did not save the original window spectra, selected bins and event-level reference annotations. A1 and A2 are D05 recordings and cannot resolve these D03-specific mechanisms. Motion contamination, transient harmonic selection, reference undercounting and synchronization error therefore cannot be distinguished. We retained all D03 pairs without causal correction or exclusion. Session-specific and rate-stratified statistics are reported in main Tables 4 and 5; the underlying numerical summaries are available from the corresponding author upon reasonable request.

S8 Acquisition timing and data completeness

A1 and A2 contain 2964 and 2959 depth frames over timestamp spans of 300.002 and 299.901 s. Their mean sampling rates, (N - 1)/span, are 9.8766 and 9.8633 Hz. The median interframe interval is 0.100 s, with maximum gaps of 0.202 and 0.402 s. Dividing frame count by the nominal 10 Hz instead gives 296.4 and 295.9 s. We used the timestamps to define acquisition time and resampled accordingly for the supplementary FFT comparison. Camera-relative time alone does not determine the reference interval boundaries.

A timekeeper coordinated acquisition and manual counting, with camera estimates concealed from the counting observer. The acquisition procedure included starting the software approximately 5 s before counting and providing a synchronization signal. A monitor-to-laptop difference of approximately 1 s concerned a different timing relationship. We did not measure the residual camera-to-reference alignment for every pair and therefore applied no uniform retrospective shift.

Table S8. Completeness of the wider study results table. Counts describe table positions, not all attempted acquisitions.

Study identifiers	Minute positions	Manual values	Camera values	Complete pairs
D01, D02, D03, D05	40	40	40	40
D04	10	10	0	0
D06	10	0	10	0
Total	60	50	50	40

Our two additional canine development image series contained 2182 and 3593 depth images with matching infrared frames, at 352 × 287 pixels. They lacked linked manual or instrumental respiratory references and were excluded from the clinical comparison. The study did not record a complete list of acquisition attempts and rejection decisions, so table completeness cannot be converted into continuous monitoring availability.

S9 Reproducibility files

The underlying paired observations, numerical waveform inputs, sensitivity-grid results and analysis code are available from the corresponding author upon reasonable request. The code supports the analyses described in this supplement. However, the original session-specific configuration and aggregation logs were not retained for every clinical minute, so exact end-to-end regeneration of all original clinical camera rates is not possible. Figure files and vector sources can also be provided upon reasonable request where available.

References

S1. Bland JM, Altman DG. Agreement between methods of measurement with multiple observations per individual. J Biopharm Stat. 2007;17(4):571-82. doi: 10.1080/10543400701329422. PMID: 17613642.

S2. Parker RA, Scott C, Inácio V, Stevens NT. Application of Mixed Effects Limits of Agreement in the Presence of Multiple Sources of Variability: Exemplar from the Comparison of Several Devices to Measure Respiratory Rate in COPD Patients. PLoS One. 2016;11:e0168321. doi:10.1371/journal.pone.0168321.

S4. Scargle JD. Studies in astronomical time series analysis. II. Statistical aspects of spectral analysis of unevenly spaced data. Astrophys J. 1982, 263, 835-853. doi: 10.1086/160554.

S5. Harris FJ. On the use of windows for harmonic analysis with the discrete Fourier transform. Proceedings of the IEEE. 1978, 66(1), 51-83. doi: 10.1109/PROC.1978.10837.
