# Supplementary material for: Technical Feasibility of Respiratory Monitoring in Dogs Using Time-of-Flight Depth Imaging
Source: Sensors (Basel). 2026 Nov 5;26(21):6986. doi: 10.3390/s26216986 (PMC13766340; doi:10.3390/s26216986)
Supplement: Supplementary file 1 [file sensors-26-06986-s001.zip › sensors-4627966-Supplementary Materials S2.pdf]

Supplementary Materials S2

ROI sensitivity of canine depth signals and spectral rate estimates

This supplement reports the effect of predefined changes in ROI size and position on the extracted depth signal and the supplementary FFT estimator. All 27 configurations in each of two D05 recordings are reported. The analysis demonstrates spatial sensitivity and does not establish respiratory-rate accuracy, interoperator repeatability or population performance.

S1 Analysis design and reproducible inputs

A1 and A2 contain 2,964 and 2,959 depth frames, respectively, with timestamp spans of 300.002 and 299.901 s. Both belong to D05. We used every depth frame, preserving the numerical TIFF values and filename timestamps. Each source image is identified by a SHA-256 checksum in Data S2. No manual count was used to select an ROI or tune a processing parameter.

We specified a new 40 x 40-pixel rectangle with zero-based columns 120-159 and rows 150-189 using the first depth images and the trunk location visible in the original ROI illustrations. This is a new baseline ROI, not a reconstruction of either original polygon. Before calculating ROI-dependent signals or rates, we fixed side lengths of 32, 40 and 48 pixels and shifts of -4, 0 and +4 pixels independently along each image axis. Their full factorial combination gives 27 configurations per recording, including the baseline. Linear size changes of 20% correspond to areas of 1,024, 1,600 and 2,304 pixels. The position shifts equal 10% of the baseline side length.

Each rectangle remained fixed for its entire sequence. Pixels with depths from 1 to 2000 inclusive contributed to the frame mean. The valid-pixel fraction was recorded at each frame. A completely invalid ROI would yield a missing signal value rather than an imputed estimate. No completely invalid ROI occurred. No window or ROI was rejected on the basis of its result.

Table S1 Fixed processing specification

Step	Specification
Time base	Filename milliseconds converted to seconds, with the first sample at zero
Resampling	Linear interpolation at 10 Hz on 0.0-299.9 s, without extrapolation
Windowing	30 nonoverlapping 10-s segments, 100 samples each
Preprocessing	Subtract each segment mean, apply a symmetric Hann window
Transform	2,000-point real FFT, exact frequencies k x 10/2000 Hz
Frequency selection	Largest magnitude within 0.10-1.50 Hz inclusive, lowest bin in an exact tie
Other filtering	No moving average, band-pass filter, quality gating or additional peak refinement
Minute aggregation	Median of six unrounded short-window rates, each frequency multiplied by 60

Step	Specification
Resolution	Native bin spacing 0.1 Hz, zero-padded grid 0.005 Hz. Zero-padding adds no independent frequency information

2 Descriptive results

The unit of biological replication remains one dog. The 27 configurations are controlled perturbations of the same images. We therefore report descriptive comparisons to the fixed baseline without hypothesis tests or population confidence intervals. Correlation and mean-subtracted RMS differences characterize similarity of depth waveforms. They are not measures of agreement with independently confirmed respiration.

Table S2 Sensitivity relative to the fixed baseline ROI

Outcome	A1	A2
Configurations, including baseline	27	27
Minimum valid-pixel fraction across every frame and ROI	98.48%	98.26%
Full-record correlation, range across 26 nonbaseline ROIs	0.914-0.999	0.884-0.999
Median 10-s correlation, range across 26 nonbaseline ROIs	0.951-0.999	0.959-0.999
Mean-subtracted RMS difference, range across nonbaseline ROIs (mm)	0.163-1.084	0.182-1.285
Mean absolute minute-estimate change, range across nonbaseline ROIs (breaths/min)	0.03-1.92	0.03-3.75
Largest absolute minute-estimate change (breaths/min)	7.50	7.80
Largest absolute 10-s estimate change (breaths/min)	17.70	30.00

For each ROI, mean absolute minute-estimate change summarizes its five differences from the baseline. Ranges across ROIs do not represent confidence intervals. Full results comprise 54 ROI summaries, 1,620 window estimates and 270 minute estimates. All are supplied in Data S2.

The baseline minute estimates were 28.50, 33.60, 32.25, 15.30 and 21.30 breaths/min for A1, and 21.60, 25.50, 28.65, 21.90 and 17.55 breaths/min for A2. These values derive from the new rectangular ROI and must not replace either the original clinical values or the previously reported estimates from the stored polygon-derived signals.

The largest minute changes occurred with the 48 x 48-pixel ROI shifted +4 pixels horizontally and +4 pixels vertically. A1 minute 5 changed from 21.30 to 13.80 breaths/min. A2 minute 4 changed from 21.90 to 14.10 breaths/min. These differences demonstrate that the minute median does not eliminate all sensitivity to the spatial extraction rule.

S3 Spectral examples and interpretation

For illustration, we selected the window-ROI combination with the largest absolute short-window rate difference in each recording after completing the full analysis. These are explicitly selected examples, not representative-window accuracy estimates. In A1 at 140-150 s, the 48 x 48-pixel ROI shifted +4 pixels horizontally and -4 vertically changed the selected frequency from 32.4 to 14.7 cycles/min. In A2 at 10-20 s, the 32 x 32-pixel ROI shifted -4 pixels in both directions changed the estimate from 36.0 to the lower search boundary at 6.0 breaths/min. The within-window waveform correlations were 0.950 and 0.996, respectively.

The spectra show changes in the relative magnitudes of competing components. Thus, a high waveform correlation can coexist with a large change in the frequency selected by the largest-magnitude rule. Without an independent respiratory-event reference, these examples do not establish which component is physiological. They also do not identify the cause of D03 discrepancies, because both recordings are from D05.

Figure S2 ROI geometry and minute estimates

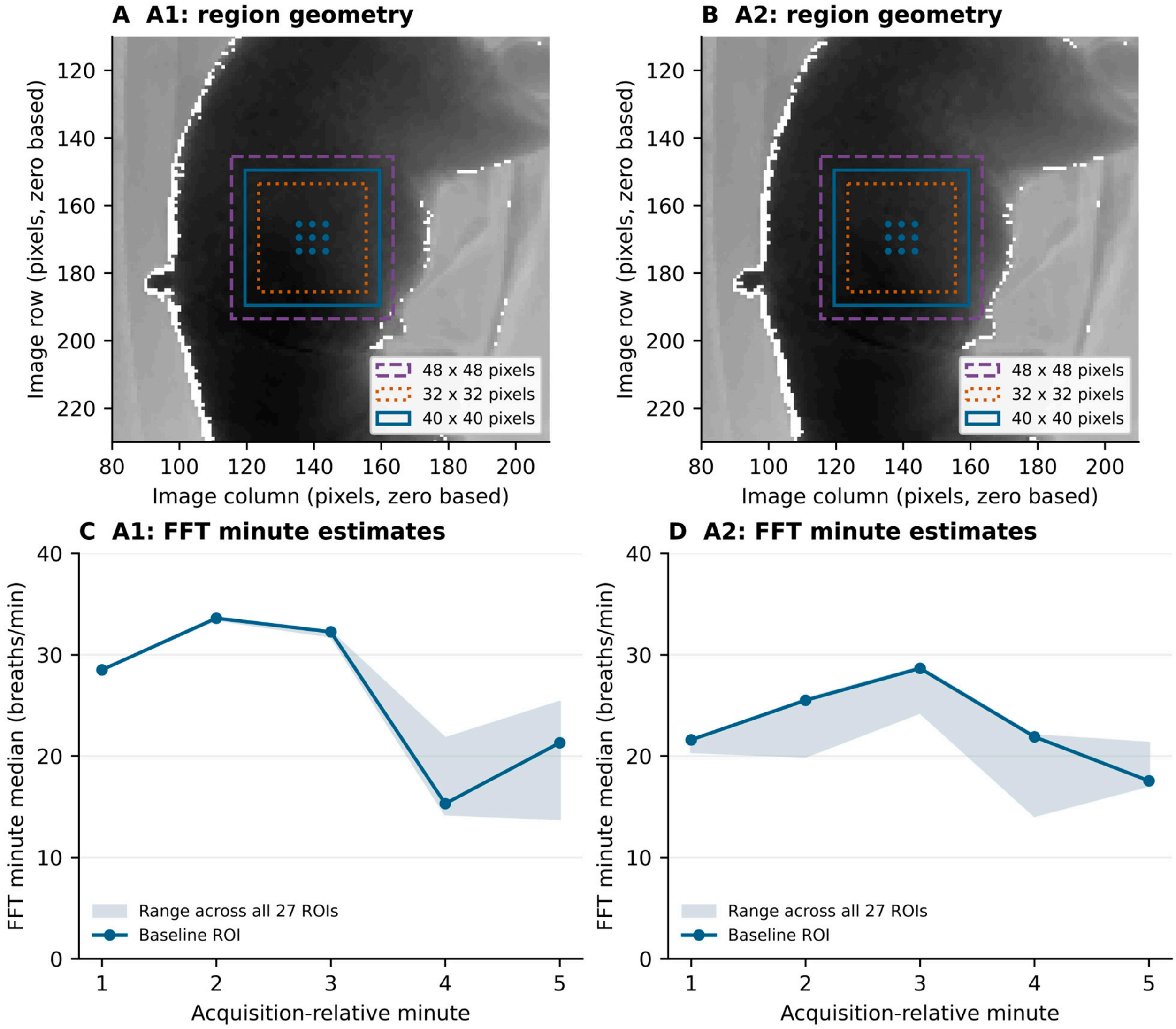

A-B: First depth images cropped around the visible trunk region. The baseline 40 x 40-pixel rectangle is blue. Centered 32 x 32 and 48 x 48 variants are orange and purple. The nine center positions indicate the shifts applied at every size, yielding 27 rectangles per recording. Coordinates refer to native image pixels. C-D: Baseline minute medians and the minimum-to-maximum envelope across all 27 ROIs. The shaded range is not a confidence interval. Lines connect acquisition-relative minute summaries. Both recordings are from D05.

Figure S3 Competing spectral components

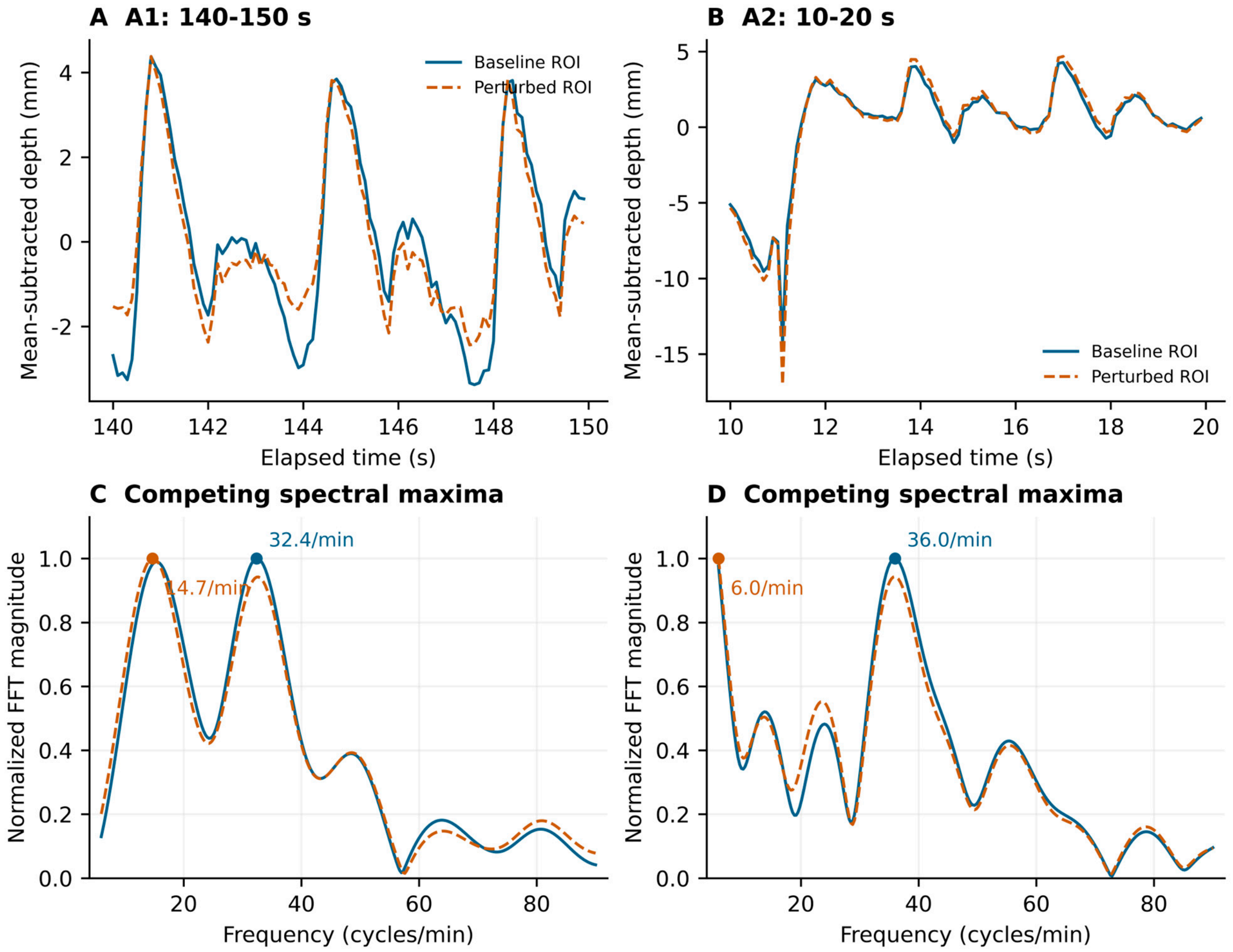

A-B: Mean-subtracted resampled depth signals in the window with the largest ROI-dependent short-window rate change in each recording. C-D: Corresponding Hann-windowed, zero-padded FFT magnitudes. Each spectrum is normalized by its own largest magnitude inside the search band. Markers show the selected maxima. A1 uses the 48-pixel ROI shifted (+4, -4) pixels. A2 uses the 32-pixel ROI shifted (-4, -4) pixels. The A2 value at 6.0 cycles/min is a search-boundary selection. These examples were selected after examining all results to illustrate spectral-selection sensitivity. Neither signal is an independent respiratory reference.

R4 Reproduction and scope

Code S2 contains the frozen ROI specification, extraction and FFT implementation, plotting script and instructions. Data S2 contains image checksums, timestamped ROI means, valid-pixel fractions and

complete output tables. Raw Depth Data S2 contains all 5,923 source depth TIFF files. A minimal example command is supplied in the package instructions. The numerical analysis used Python 3.12 and NumPy 2.3.5. Raster figures were exported at 1200 dpi, with vector versions supplied.

The original polygon masks and exact alignment to manual minute boundaries are not recovered by this experiment. The 40 clinical pairs remain an exploratory comparison of the values recorded during the study. This supplement provides no additional independent dogs, no breath-event validation, no repeated observer counts and no denominator of all attempted acquisitions.
